# Exploration of biomaterial-tissue integration in heterogeneous microporous annealed particle scaffolds in subcutaneous implants over 12 months

**DOI:** 10.1016/j.actbio.2025.02.020

**Published:** 2025-02-14

**Authors:** Ethan Nicklow, Lauren J. Pruett, Neharika Singh, James J. Daniero, Donald R. Griffin

**Affiliations:** a Department of Biomedical Engineering, University of Virginia, 415 Lane Road, Rm 1213, Charlottesville, VA 22903, USA; b Department of Otolaryngology-Head and Neck Surgery, University of Virginia; Charlottesville, Virginia 22903 USA; c Department of Biomedical Engineering and the Department of Chemical Engineering, University of Virginia, 415 Lane Road, Rm 1111, Charlottesville, VA 22903, USA

**Keywords:** Porous hydrogel, MAP scaffold, Tissue formation, Inflammation, long-term

## Abstract

Microporous annealed particle (MAP) scaffolds are comprised of hydrogel microparticles with inter- and intra-particle cross-links that provide structure and cell-scale porosity, making them an increasingly attractive option for injectable tissue augmentation. Many current injectable biomaterials create a substantial foreign body response (FBR), while MAP scaffolds mitigate this response and have the potential to facilitate the formation of new tissue, though this *de novo* tissue formation is poorly understood. Here, we leverage a subcutaneous implant model to explore the maturation of MAP implants with and without heparin microislands (μislands) over one year to identify the effect of bioactive particles on scaffold maturation. Implants were measured and explanted after 1, 3, 6, and 12 months and analyzed using immunofluorescence staining and RNA-sequencing. No fibrous capsule or significant FBR was observed, and though a significant amount of MAP remains at 12 months, we still see a volume decrease over time. Heparin μislands facilitate increased cell infiltration and recruit a wider variety of cells at 1 month than blank MAP scaffolds, although this effect diminishes after 3 months. Transcriptomics reveal a potential activation of the complement-mediated immune response at 12 months in both groups, possibly associated with pore collapse in the implants. A single 12-month sample avoided this outcome, yielding complete cell infiltration, vascularization, and substantial matrix deposition throughout. Future work will characterize the effect of implantation site and facilitate increased matrix deposition to support the scaffold and prevent pore collapse.

## Statement of significance

Injectable biomaterials are increasingly used clinically for soft tissue augmentation and regeneration but still face significant issues from the foreign body reaction. While some materials intentionally promote this response to stimulate collagen deposition, porous materials like MAP scaffolds can mitigate the immune response and allow for true tissue integration. However, this integration is poorly understood, particularly on long timescales, as traditional materials are dominated by inflammatory signals. In this work, we leverage a minimally inflammatory subcutaneous implant to investigate the maturation of MAP scaffolds with and without bioactive heparin-containing particles. The results presented here contribute a better understanding of the long-term material-tissue dynamics of MAP scaffolds that can inform future material design for tissue augmentation.

## 1 Introduction

Clinical use of injectable biomaterials has grown popular for soft tissue bulking in the last 30 years; although it was initially motivated by needs in plastic surgery, the advent of materials engineered for long-term, stable tissue augmentation has greatly broadened the possible applications [1–5]. However, the implantation of these materials initiates an acute immune response that can develop into a chronic response called the foreign body response (FBR). The FBR is characterized by sustained inflammation around the implant and fibrous encapsulation, both of which can accelerate implant degradation and detrimentally affect the surrounding tissue. In addition, this response makes biomaterial development complicated as inflammation dominates the tissue response, particularly impacting the study of long-term interactions between material and tissue. However, many materials currently in clinical use, including Radiesse (calcium hydroxyapatite microspheres in carboxymethylcellulose carrier gel) or Silk Voice (silk particles in hyaluronic acid carrier gel) [6,7], employ strategies to promote the FBR, stimulating chronic inflammation with associated collagen deposition that creates tissue bulk via a sustained FBR and fibrotic response [3,8]. Alternatively to this pro-fibrotic approach, there is substantial evidence that introducing porosity into a material can affect the immune response and mitigate the FBR [9–11]. The microporous annealed particle (MAP) scaffold system is one such porous material, comprised of a slurry of hydrogel microparticles that undergo both intra- and inter-particle cross-linking to form a stable scaffold with cell-scale porosity. Previously, MAP scaffolds have achieved superior outcomes for skin regeneration in wound healing on short timescales and vocal fold augmentation on longer timescales compared to commonly used clinical standards (Oasis wound matrix and Restylane, respectively) [10,12–14].

Many of these studies utilize a heterogeneous MAP scaffold system (heparin μislands) with 10 % of particles containing heparin, leveraging its endogenous growth factor sequestration [13,15,16]. This system has been shown to accelerate dermal wound healing outcomes and cell migration into MAP implants on short timescales, using a degradable microparticle formulation containing heparin μislands [13]. Recent studies using non-enzymatically degradable MAP scaffolds for vocal fold augmentation (VFA) have demonstrated success for up to 14 months [12], providing tissue volume longer than the clinical standards and facilitating *de novo* tissue formation in most samples. This tissue formation is particularly interesting, given VFA materials ideally provide permanent augmentation; however, some commonly used clinical materials intentionally degrade (i.e., Restylane), allowing for ingrowth but failing to provide long-term tissue support [10,12–14]. Other materials lack an engineered degradation mechanism (i.e., Radiesse, Silk Voice) and remain present in the tissue for long periods of time, relying on stimulated collagen deposition for bulking [6,7]. The observed *de novo* tissue was collagenous and did not significantly differ in fibrotic content compared to the contralateral control sample. However, this study only examined samples *ex vivo* after 14 months, limiting any mechanistic understanding of this tissue formation. Therefore, we were interested in studying the dynamics of long-term tissue integration in more temporal and spatial detail in a simpler implant model. We hypothesize that non-enzymatically degradable MAP scaffolds undergo multiple phases of cellular infiltration and activity, eventually leading to the deposition of matrix proteins that will support implant structure as the microparticles degrade.

In addition, we wanted to leverage the modular nature of MAP scaffolds to explore the timeline of tissue integration by comparing MAP scaffolds with and without bioactive heparin μislands. We have previously shown that heparin μislands can harness endogenous bioactivity to accelerate tissue formation [12,13] without impacting inflammatory response, but the bioactivity and lifetime of effect of the heparin μislands are not well characterized. To simplify the *in vivo* model and isolate the scaffold bioactivity as our primary variable, we chose to implement a minimally inflammatory subcutaneous implant model for up to 12 months, as this would allow for an exploration of how, in the absence of significant inflammatory signals, the surrounding tissue interacts and integrates with scaffold. We hypothesize that the heparin μislands will drive accelerated cell migration and tissue formation within the scaffold without significantly impacting the FBR.

## 2. Methods

### 2.1. Heparin modification

Heparin was modified to contain a thiol-group as previously described [13], allowing it to participate in the pseudo-Michael addition cross-linking of the microparticles. Briefly, heparin was reacted with 100 mM 3-(2-pyridyldithiol) propionyl hydrazide (PDPH) with a target of modifying 20 % of the heparin repeat units. Each day for three days, the pH was adjusted to 6.5,then the solution was reacted with4-(4, 6-Dimethoxy-1,3,5-triazin-2-yl)–4-methylmorpholinium (DMTMM) at 1:1 molar ratio to the heparin repeat units. This solution was then dialyzed in 1 M NaCl followed by 0.01 M NaCl, then frozen and lyophilized. After weighing the lyophilized product to estimate purity, the product was reconstituted in MilliQ water and deprotected with 25 mM TCEP, then dialyzed in 0.01 % trifluoroacetic acid and 1 M NaCl (followed by dialysis in decreasing salt concentrations for several hours), then finally frozen and lyophilized again. The final product was stored at −20 °C until use. Heparin thiolation was quantified using previously established methods [13] using a pyridine-2-thione assay in conjunction with NMR.

### 2.2. Microgel fabrication

#### 2.2.1. Materials

4-arm poly(ethylene glycol) (PEG) maleimide (PEG-MAL, 10 kDa and 20 kDa) and 4-arm PEG thiol (PEG-SH, 10 kDa) were purchased from Nippon Oil Foundry, Inc (Japan), and RGD cell adhesive peptide (Ac-RGDSPGGC–NH_2_) were obtained from WatsonBio. Materials were aliquoted after dissolution in MilliQ H_2_O for ease of use and repeatability. A custom annealing macromer, MethMal (4-arm PEG-maleimide modified to have 2–3 methacrylamide arms), was synthesized as previously described [17]. The inclusion of MethMal allows for the secondary cross-linking reaction (referred to as annealing here) to occur between methacrylamide groups on adjacent particles, decoupled from the initial thiol-maleimide crosslinking.

#### 2.2.2. Formulations

Heparin-containing particles were synthesized at 1.6 % (w/v) with 3 mg mL^−1^ heparin incorporation. Particles without heparin were synthesized using the same methods at 2.3 % (w/v). Final concentrations for heparin-containing particles were: 32.40 mg mL^−1^ PEG-maleimide, 17.09 mg mL^−1^ PEG-SH, 1 mM RGD, 1 mM MethMal, and 5μM biotin-maleimide. Final concentrations for particles without heparin were: 34.67 mg mL^−1^ PEG-maleimide, 23.4208 mg mL^−1^ PEG-thiol, 1 mM RGD, 1 mM MethMal, and 5μM biotin-maleimide.

#### 2.2.3. Particle generation

Parallel step-emulsification microfluidic devices designed by Rutte et al. [18] with a channel height of 14.5 μm were used to generate particles following previous protocols [13,19] for microfluidic particle generation. Briefly, maleimide-containing components were dissolved in 10x PBS pH 1.9, and thiol-containing components were dissolved in 1x PBS pH 7.4. The solutions were combined 1:1 then loaded into a syringe to run through the microfluidic device. Surfactant (Ran Biotechnologies) was diluted to 2 % (w/v) in NOVEC-7500 (3M) oil for use in the device. Syringe pumps were run at 3 mL hr^−1^ for the aqueous solution and 6 mL hr^−1^ for oil. Particles were allowed to cross-link overnight before purification via serial washes and transitions into PBS. Particles were incubated with a 100 mM n-acetyl-cysteine solution to quench unreacted maleimide groups. Finally, particles were sterilized via serial washes with 70 % isopropanol in a biosafety cabinet followed by storage at 4 °C.

#### 2.2.4. Formulation characterization

Microparticles were reacted with FITC-conjugated streptavidin to tag them via streptavidin/biotin binding. The particles were then imaged using a confocal microscope at 10x magnification and analyzed using a custom ImageXpress macro to output particle diameter measurements. Particle size can be modulated by using microfluidic devices with varying channel heights, adjusting the relative flow rates of oil and aqueous solutions, and changing the polymer weight fraction. The formulations were also used to create macrogels for characterization via compression testing as well as microparticles. Briefly, 100 uL of the aqueous gel precursor solution was pipetted onto slides coated with Sigmacote^®^ and 2 mm rubber spacers, allowed to react for 24 h, then placed in 1X PBS to swell overnight. These macrogels are solid, nanoporous cylinders of the same gel formulation that comprises the MAP microparticles, as it has previously been shown that compressive testing of macrogels matches mechanical properties of microparticles [20]. Macrogels were tested using a 5 N load cell on an Instron mechanical tester with a strain rate of 1 mm min^−1^. A Matlab (Mathworks, R2022b) script was used to analyze the slope of the stress-strain curve to estimate the elastic modulus.

#### 2.2.5. Heparin incorporation

An input concentration of 3 mg/mL heparin was used in the heparin-containing formulation. To test the final concentration of heparin, heparin was tagged with Alexa Fluor 555 during the modification process (via incorporation of Alexa Fluor 555-hydrazide) during the initial PDPH reaction step) then imaged along with a standard curve to estimate the concentration of heparin on the particles [13].

#### 2.2.6. Syringe preparation

Prior to injections, particles without heparin (MAP) were mixed with heparin-containing particles at a 10:1 ratio to form 10 % heparin μislands MAP. Both 10 % heparin μislands MAP and standard MAP were mixed 1:1 volumetrically with 40 μM Eosin Y as a photoinitiator, incubated overnight at 37 °C, then loaded into syringes the day of injections (and kept out of light) using positive displacement pipettes in a biosafety cabinet. Eosin Y was chosen as it initiates in green light rather than UV light, and higher wavelengths penetrate tissue better than shorter ones [21,22].

### 2.3. Subcutaneous implants

#### 2.3.1. Implantation

All animal work was conducted with the approval of the University of Virginia Animal Care and Use Committee under Protocol #4165. 8-week-old female Swiss Webster mice were obtained from Charles River. The mice were anesthetized with isoflurane mixed with oxygen before all procedures. One day before subcutaneous injections, hair was removed from a section of the dorsal side of each mouse via shaving and hair removal product (Nair). On the day of injections, the skin was cleaned with alcohol wipes. Using forceps, the skin was gently pulled up, and a 25 gauge needle was inserted subcutaneously to inject approximately 100 μL of MAP or 10 % heparin μislands MAP. To anneal the scaffolds of microparticles, a green LED (505 nm) was used at 180 mA for 75 ss, as previous testing with mouse skin showed this yielded the required irradiance of 866 mW cm^−2^ to initiate the annealing reaction via Eosin Y. This matches the total energy delivered in previous studies [17] by decreasing time but increasing intensity. Implants were measured with calipers immediately following injection according to previous protocols [14]. Mice did not receive an analgesic, as this implantation procedure is approved under USDA Pain and Distress Category C.

#### 2.3.2. Harvesting

Mice were sacrificed at 1, 3, 6, and 12 months according to standard protocols via isoflurane overdose and cervical dislocation. Block molds were prepared with labels and placed on dry ice. Implants were removed with surgical scissors, keeping the layer of skin on top of the implants but removing any fascia still attached. The implants were immediately placed on pre-frozen optimal cutting medium (OCT) in the labeled blocks and frozen on dry ice, then stored at −80 °C.

### 2.4. Tissue histology and immunostaining

#### 2.4.1. Hematoxylin and Eosin (H&E) histology

H&E staining was performed using previously published protocols for frozen tissues [12].

#### 2.4.2. Tissue section immunofluorescence

OCT blocks were sectioned at 10–20 μm with three sections per slide, each at least 150 μm apart. Details for each antibody and staining condition are detailed in the table below. For staining, slides were first fixed in the appropriate solution (specified in Table S1). Slides were rinsed in 1X PBS (2 × 5 min) then 0.3 % (v/v) TritonX-100 in 1X PBS (PBS-T) (1 × 5min). Blocking was performed with the appropriate buffer (specified in Table S1) for 30 min at room temperature. After blocking, slides were incubated with the primary antibody prepared in 1 % serum diluted in PBS-T. The serum for each stain set corresponds to the animal the secondary antibodies were raised in (i.e., goat, donkey). Primary antibodies were incubated overnight at 4 °C. Washing was performed with 1 % serum in PBS-T (2 × 5 min) prior to secondary antibody incubation. Secondary antibodies were diluted in 1 % serum in PBS-T or just PBS-T for 1–2 h at room temperature. All secondary antibodies were purchased from Invitrogen with conjugated fluorophores. Slides were then washed with 1 % serum in PBS-T (1 × 5min) followed by just PBS (2 × 5 min). If autofluorescence quenching was required, the VectorLabs TrueView autofluorescence quenching kit was used with a 4 min incubation time. Slides were washed in PBS, incubated in DAPI (1:200 if TrueView was used, 1:1000 otherwise) diluted in 1X PBS for 20 min at room temperature. Slides were washed one final time, then mounted with VectorLabs TrueView mounting medium or Prolong Gold anti-fade mounting medium.

### 2.5. Image analysis methods

All implant sections on each slide were imaged using a Zeiss Axioscan 7 slide scanner with a 20x objective to visualize all stains with their associated secondary antibody conjugate. After imaging, images were exported and cropped using the DAPI channel to isolate the implant from the surrounding skin and fascia.

#### 2.5.1. Extracellular matrix (ECM) analysis

For analysis of DAPI, collagen I, collagen III, collagen IV, and fibronectin staining, a custom ImageJ macro script was written to threshold the images (using the algorithm determined to be best for each stain via comparison to negative controls), iteratively erode the implant region of interest (ROI) by 100 μm, and export fraction of positive pixels for each band of ROI (see Figure S2). This process allowed for data on the effect of distance from the edge of the implant as well as time and heparin inclusion and was analyzed as outlined in Statistical Analyses.

#### 2.5.2 Endothelial cell analysis

For analysis of CD31 and podoplanin, the ImageJ *Analyze Particles* function was used, along with Otsu’s thresholding method, to threshold and count the number of CD31^+^ and CD31^+^ Podo^+^ cells, normalized to the area of each implant. Using a similar method as in ECM analysis, a custom Matlab (Mathworks) script was written to erode the implant ROIs to analyze differences between the core of the implant and the outer region, with a cutoff determined by the radius that would yield 50 % of the area.

#### 2.5.3. Macrophage, T-cell, fibroblast, and Ki67 analysis

For analysis of macrophages and T-cells in Fig. 4, the imaging processing software IMARIS (Oxford Instruments) was used for background subtraction and object-based colocalization. Briefly, the Spots function was used to create spots for all DAPI signal (representing individual cells), as well as CD68 staining (representing all macrophages), iNOS staining (M1), and Arg-1 staining (M2). The spots were iteratively filtered based on the distance between spots (greater than 8 μm to distinguish individual cells) and the distance to markers of interest. For example, M1 macrophages would be filtered as follows: DAPI spots within 8 μm of a CD68 spot, filtered by distance to iNOS spot (<8 μm). Total cell number, total macrophage number, and M1/M2 numbers were all recorded for macrophages. T-cell analysis followed a similar process, first filtering by CD3e^+^ spots (all T-cells) and then focusing on CD4^+^ spots (T-helper cells). Percentages were calculated as well as cell counts normalized to area.

A similar process was used to analyze Ki67 staining for proliferation, with spots created for both the DAPI channel and the Ki67 channel. The DAPI spots, representing cells, were filtered by distance from a Ki67 spot to ensure they were effectively overlapping, then counted to yield the fraction of Ki67^+^ cells.

Finally, to analyze fibroblast marker (TE-7) staining, the Spots function was used to identify all cell nuclei, and the Surfaces function was used to create boundaries for all TE-7 staining. Fibroblasts were identified as DAPI Spots within 2 μm of a TE-7 surface and quantified both as % of total cells and cells normalized to the implant area.

### 2.6. RNA-seq

#### 2.6.1. RNA isolation and sequencing

After consultation with the UVA BTRF core, we modified the standard protocol for Micro RNeasy Kits (Qiagen) for RNA isolation from laser-dissected microsections to isolate RNA from flash-frozen, OCT-embedded implants. Labeled tubes were filled with Buffer RLT and put on ice. OCT blocks were sectioned at −25 °C with a cryostat and sections were moved from the cryostat plate directly into the tubes on ice. The cryostat blade and forceps used to move sections were cleaned with RNase Zap^®^ prior to use to prevent RNA degradation. The remaining steps closely follow the protocol for Purification of Total RNA from Microdissected Cryosections from the Qiagen Micro RNeasy Kit (Qiagen). After RNA was quantified and purity was assessed using a Nano-Drop (Thermo Scientific) and an Agilent RNA ScreenTape Analysis Kit on an Agilent 4200 TapeStation (via the UVA GATC), samples were processed for library preparation using the NEBNext Ultra II Directional RNA Library Prep Kit (Illumina), according to validated standard operating procedures established by the University of Virginia Genome Analysis and Technology Core (GATC), RRID:SCR_018883. cDNA quality was assessed with an Agilent High Sensitivity D5000 ScreenTape analysis kit then sequenced on an Illumina NextSeq 2000 with a P2–100 flow cell.

#### 2.6.2. RNA-seq analysis

All data was downloaded from Illumina BaseSpace as zipped FASTA files. We used Kallisto [23], a pseudo-alignment and quantification algorithm, to align and quantify paired-end reads for each sample to a reference M*us musculus* transcriptome downloaded from Ensembl (GRCm39). Each sample had 50–80 % reads aligned and over fifteen million reads mapped. Alignment was done automatically using a custom Powershell script via Kallisto. After quantification, differential gene expression analysis was performed with Sleuth [24], a companion R package that can read Kallisto outputs and leverage the bootstraps it produces to estimate technical variation. Batch and mouse-to-mouse variability were accounted for as random-effects in the linear mixed model, with heparin μislands (Treatment) and time (Timepoint) also included as predictors. A likelihood ratio test (LRT) was performed to identify genes with variation better explained by the predictors rather than the random effects. Subsequent Wald tests were performed on various contrasts between specific to identify differences in expression between conditions. The *removeBatchEffects* method from the limma package [25] in R was used to remove batch effects for visualization in the PCA analysis while preserving variation associated with timepoint and treatment. Pathway analysis was performed using the Fast Gene Set Enrichment Analysis (FGSEA) package in R [26] and the Reactome canonical pathways database. Pathways were filtered with an adjusted p-value threshold of 0.01 and collapsed to avoid redundant pathways. All code is available upon request.

### 2.7. Statistical analyses

Aside from the spatial analyses in Figs. 2b-c, 3b-c, 3e-f, 3h-i, and 3k-l, all statistical tests and visualizations were performed in GraphPad Prism 10.2.0. Unless otherwise noted, all multi-group analyses were two-way ANOVA with matched samples (mouse-to-mouse controls) and multiple comparisons corrected using Tukey tests. P-values greater than 0.05 were considered significant. Graphs are represented with mean ± standard deviation.

#### 2.7.1. Spatial analysis

To analyze the effects of our predictor variables on distance trends, data from the image analysis methods was imported into R and fit using linear mixed models (*lmer* package in R [27]), accounting for variability from each mouse, image replicate, group (heparin/no heparin), timepoint, and distance from implant edge. The final model accounted for the variability from each mouse within the groups and any variability in image replicates as random effects and group, timepoint, and distance (as well as their interactions) as linear effects. The *emmeans* and *emtrends* packages in R were used to analyze the results with pairwise comparisons between each group at each timepoint and ggplot2 was used to visualize the resultant models. Each line includes a 95 % confidence interval in grey. The code is available upon request. Statistical advice was given by statisticians in the Public Health Sciences department in the University of Virginia Health Sciences Library (UVA HSL).

## 3. Results

### 3.1. Isolating heparin μislands by matching particle properties

**Fig. 1.**
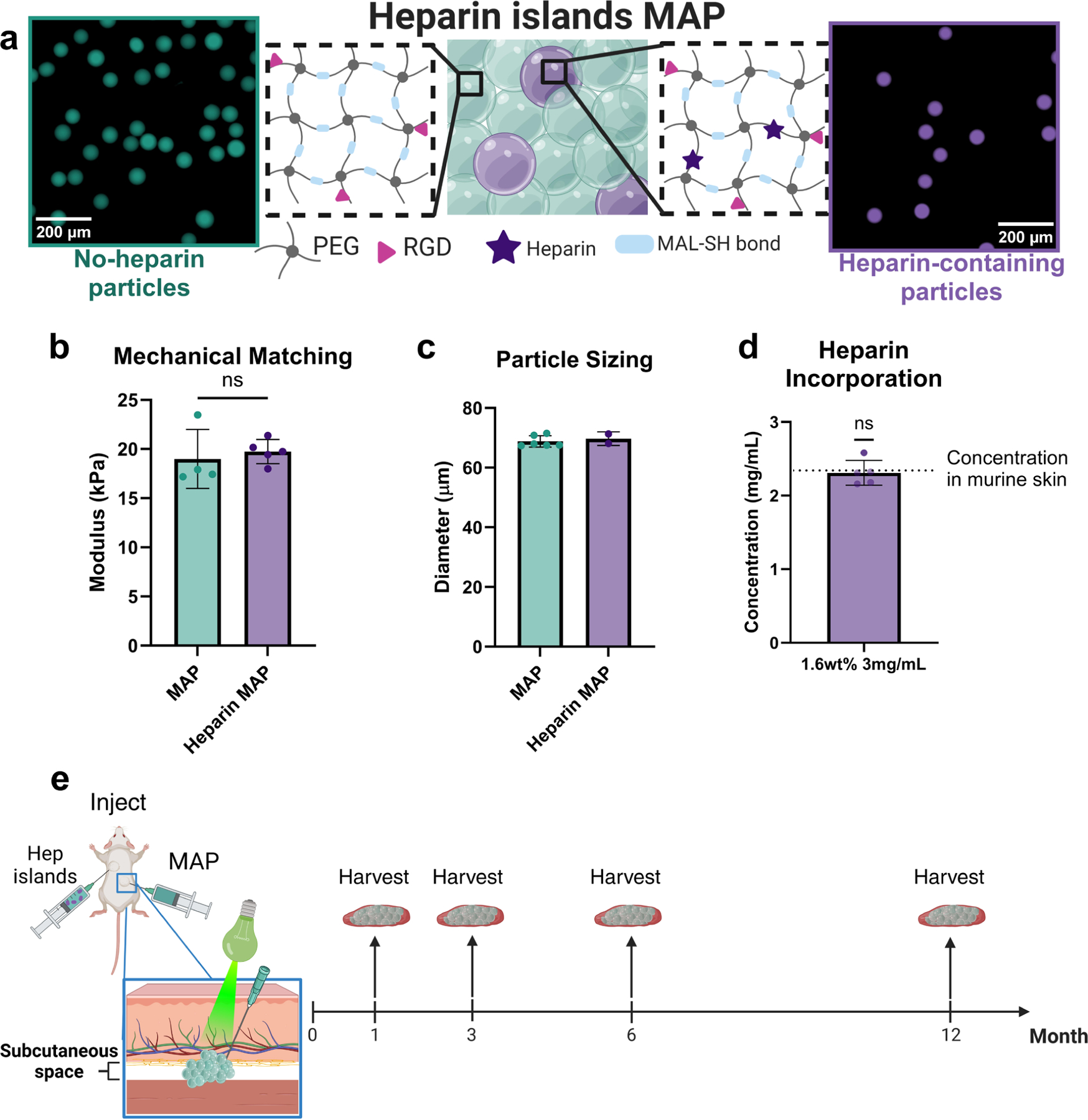
Material characterization and study design. **a)** Both particle formulations use similar backbones comprised of 4-arm 10 kDa PEG-maleimide and PEG-thiol that participate in Michael-type addition reactions. RGD cell adhesion peptides and heparin are incorporated via thiol linkages. Heparin-containing particles are mixed with non-heparin particles at 10 % v/v. **b)** Particle formulations were mechanically tested via compression testing and compared with a student’s *t*-test. **c)** Particles were sized via confocal microscopy to ensure they were matched in size. **d)** Final heparin concentration was assessed via the fluorescent tag incorporated during thiolation. With an input concentration of 3 mg mL^−1^, a final concentration of 2.3 mg mL^−1^ was achieved matching endogenous levels in skin (dotted line). **e)** Graphic describing study design and relevant timepoints. An initial *n* = 6 mice were designated for each timepoint, but given the long nature of the study, those were altered for each timepoint as follows: 1 month, *n* = 6; 3 months, *n* = 5; 6 months, *n* = 3; 12 months, *n* = 6. Implants that had merged, migrated, or otherwise appeared altered were excluded. The zoomed in diagram (not to scale) shows a schematic of the injection and light-based annealing process. Created in part with Biorender (2024).

Using previously established methods [13,17], we generated particles with and without heparin that were matched in size and stiffness as determined by confocal microscopy and compression testing, respectively (Fig. 1b-c). Particles were generated with and without heparin (Fig. 1a) and size-matched (diameters of 69.7 ± 2.25 μm and 68.8 ± 1.87 μm, respectively), as particles of similar sizes in scaffolds of approximately equivalent porosity will yield similar pore dimensions [28] which has been shown to affect cell behavior in a variety of ways [20,29,30]. By matching local stiffness and pore architecture, both key factors in determining cell behavior, we were able to isolate the inclusion of heparin-containing particles (heparin μislands) as the only variable changed between the two conditions over time. Heparin content in these particles was matched to previously determined endogenous heparin levels in mouse skin [13] (Fig. 1d), which was deemed most appropriate given the subcutaneous environment. Particles were mixed to achieve a 1:10 ratio of heparin-containing particles to total particles (10 % heparin μislands).

### 3.2. Implants show extensive cell infiltration and retain some volume to 12 months

**Fig. 2.**
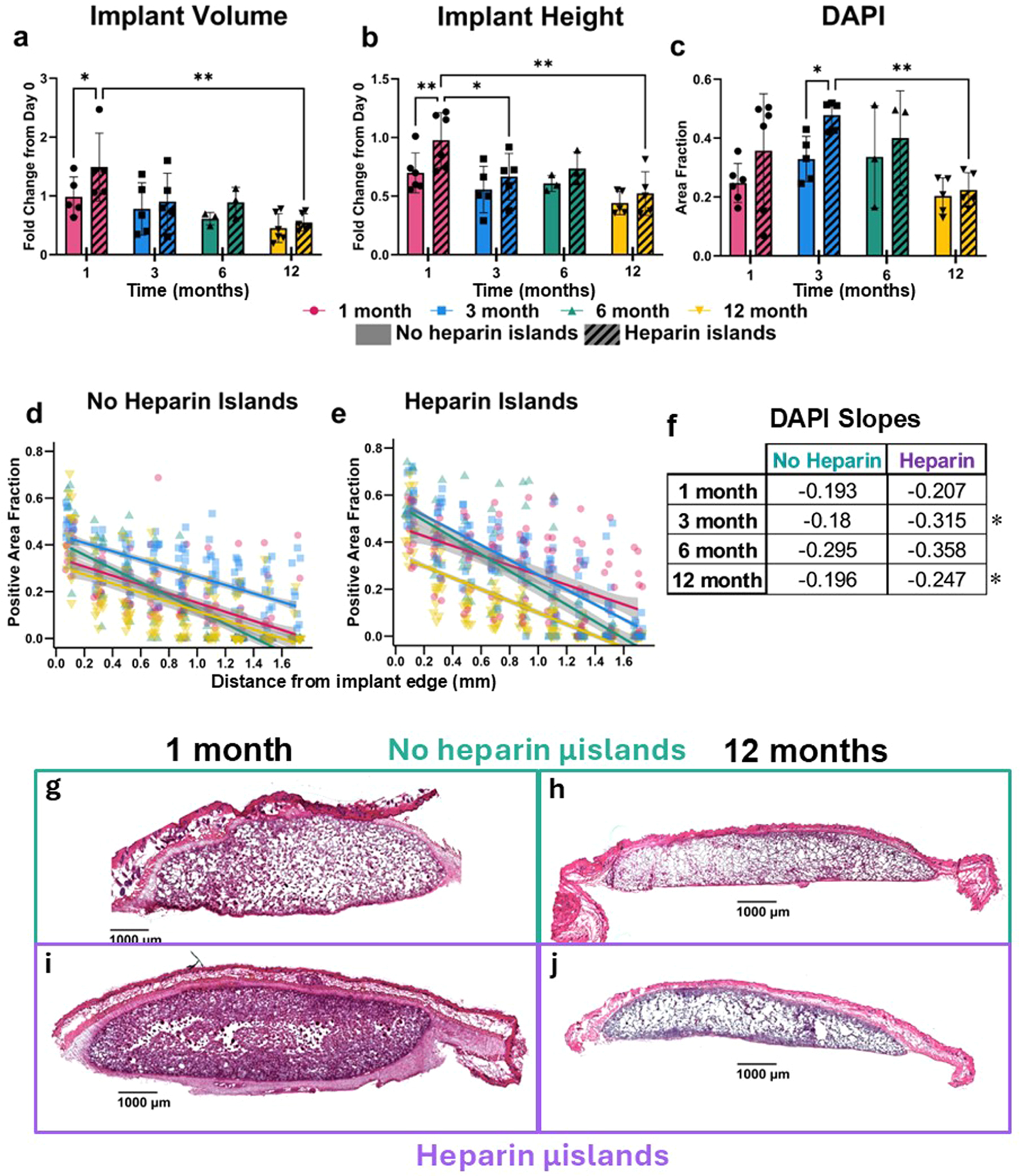
Implant stability and integration. The dimensions of each implant were measured with calipers immediately after implantation and before harvesting. Volume fold change **a)** and height fold change **b)** were both calculated to track changes over time. **c)** Cell infiltration was assessed via the percent of positive pixels for DAPI staining, as individual cells were often difficult to separate in the tightly packed pores. As described in the methods, spatial data was obtained and analyzed for the DAPI staining for both Hep samples **d)** and NoHep samples **e)**. The slopes with respect to Distance were calculated using *emtrends*. Stars represent *p* < 0.01 when comparing the slopes of each group for each timepoint. **f)**. Representative images of H&E-stained implants **g) – j)** are shown; all scale bars are 1000 μm (1 mm).Images were white balanced then cropped with no further image adjustments. All statistical tests in this figure are two-way repeated measures ANOVAs with Tukey post-hoc testing. **a)** – **c)** *p < 0.05, **p < 0.01, ***p < 0.001, ****p < 0.0001. Error bars are mean ± SEM per group. Outlier analysis: Outliers from any of the datasets were removed using the Robust Regression and Outlier Removal (ROUT) method (*Q* = 1 %) in GraphPad Prism.

Implants were first evaluated for stability in a subcutaneous environment for one year to validate that this non-enzymatically degradable formulation would avoid significant degradation via other mechanisms (i.e., reactive oxygen species, hydrolysis), given this study is the longest to date for subcutaneous implantation of MAP scaffolds (previous longest was 4 months [14]). The dimensions of each implant were externally measured with calipers immediately following implantation and prior to harvesting, allowing for fold-change calculationsn (Fig. 2a-b). Initial measurements were also analyzed, and no significant differences were observed (Figure S3).

Both groups (Hep and NoHep) had similar volume and height fold changes over time, although Hep samples maintained the initial height out to 1 month. Regardless, both groups saw an approximately 55 % decrease in volume at 12 months, indicating these implants are stable but still experience a reduction in volume over time. Analysis with 2-way ANOVAs show a significant effect due to both heparin island inclusion and time for implant volume and implant height. Despite this eventual volume decrease, there is extensive cell infiltration into the implants, peaking at 3–6 months in both groups (Fig. 2c). Hep samples see increased cell presence at 3 months as well, suggesting a chemotactic effect that has been corroborated in previous studies [13]. However, after 12 months, there is a reduced cell presence in both groups that accompanies the decrease in implant volume; this contrasts with the results seen in Pruett et al. in a vocal augmentation context [12].

Looking into the spatial cell distribution, we segmented the implant regions of interest (ROI) into concentric rings (Figure S2), allowing for spatial data over time (Fig. 2d-f). We see a significantly (*p* ≤ 0.002) less step gradient at 1 month in Hep samples compared to later timepoints, indicating an increase in cells within the middle of the implants and more uniform infiltration. Additionally, all Hep samples after 1 month have significantly (*p* ≤ 0.05) steeper spatial gradients than NoHep samples, suggesting that Hep samples continually encourage cell migration into the edges of the implant but are unable to facilitate deeper infiltration. At longer timepoints (6 and 12 months), both groups have similar total cell infiltration (Fig. 2c), but Hep samples tend to have steeper gradients (Fig. 2f, Table S2), suggesting that cells congregate closer to the implant edge compared to NoHep samples.

### 3.3. Heparin μislands facilitate increased endothelial infiltration and increased cell proliferation at 1 and 12 months

**Fig. 3.**
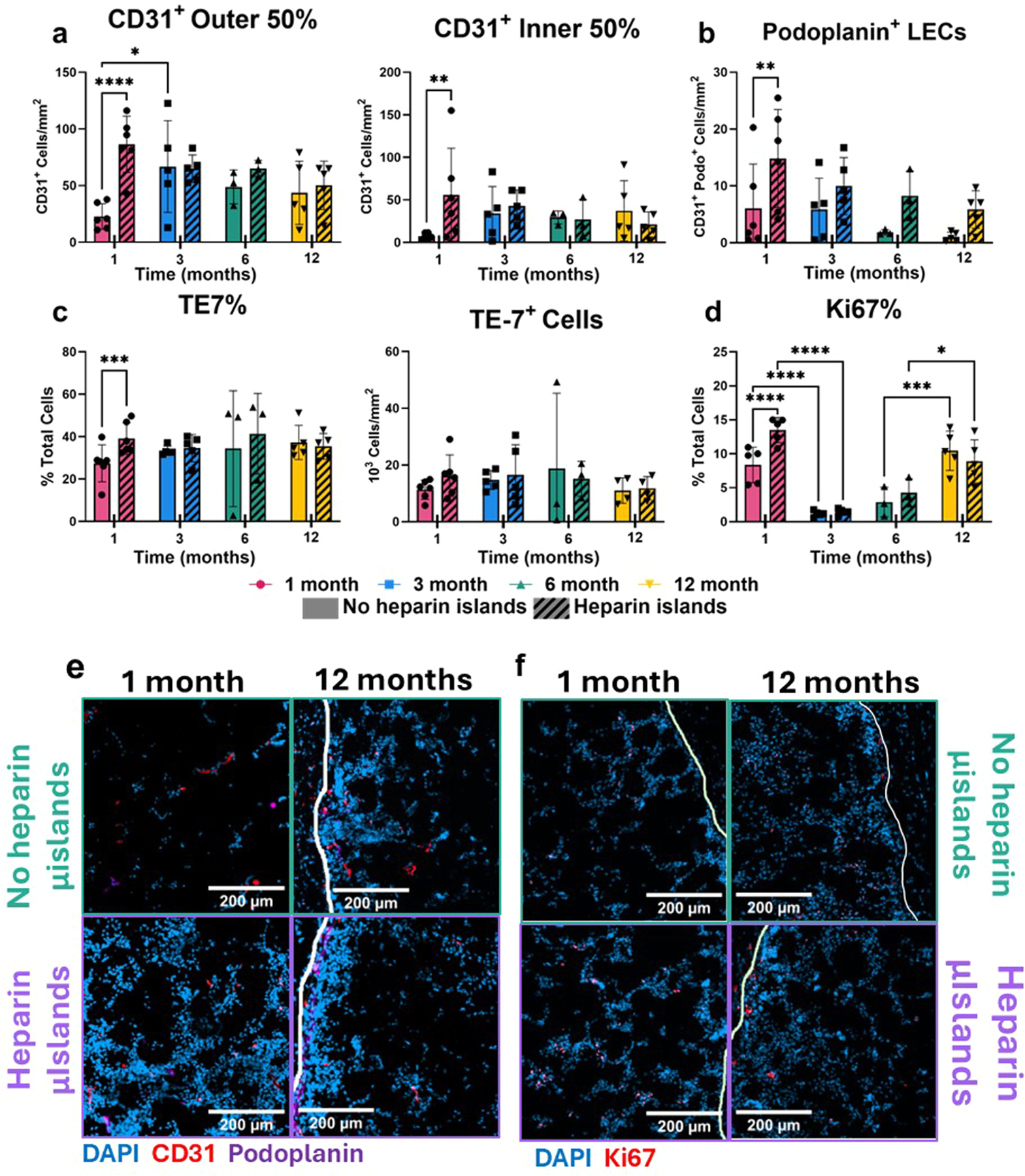
Stromal cell infiltration. Each implant was segmented into an outer and inner region, with each region comprising 50 % of the total area. CD31^+^ cells were counted in each region **a, e)** using a custom Fiji script and normalized to implant area. **b, e)** Lymphatic endothelial cells (LECs) were also counted using a similar method after filtering for colocalization with podoplanin. **c)** Fibroblasts were quantified in terms of the % total cells and cells/area for each implant using the TE-7 marker. **d, f)** Proliferation was assessed via Ki67 nuclear expression. Not all pairwise comparisons are shown for **d)**. Representative images were chosen from samples closest to the mean for each graph to ensure an accurate representation of the data. All scale bars are 200 μm and any white lines on images represent the edge of the implant. All statistical tests in this figure are two-way repeated measures ANOVAs with Tukey post-hoc testing. *p < 0.05, **p < 0.01, ***p < 0.001, ****p < 0.0001. Error bars are mean ± SEM per group.

To investigate implant vascularization, especially given known interactions between heparin and vascular endothelial growth factor (VEGF), we stained implants with CD31, a marker for endothelial cells (ECs), and podoplanin, a marker associated with lymphatic ECs [31]. We see a significant increase in CD31^+^ cells in Hep samples (Fig. 3d-e), with a particularly strong effect within the inner 50 % of the implant (Fig. 3e) as seen previously with heparin μislands MAP [13]. Included among the ECs recruited are lymphatic endothelial cells (LECs), marked as CD31^+^ Podo^+^ cells, which see a similar increase in Hep samples.

Looking at fibroblast infiltration and cell proliferation, heparin μislands inclusion leads to an increased fraction of TE-7^+^ cells and increased proliferation at 1 month, measured via nuclear Ki67 expression. Fibroblast cell fraction changes little over time, with the effect of heparin μislands diminishing by 3 months. The fraction of proliferating cells (Ki67^+^%) is higher at 1 month in both samples compared to 3 and 6 months, followed by a spike at 12 months. These results indicate a period of inactivity after the initial infiltration into the implant that ends around 12 months after implantation. Despite these positive results early in the implant lifetime, the impact of the heparin μislands across all cell types here diminishes as early as 3 months, with a general decreasing trend over time for both groups in terms of cell presence, vascularization, and lymph vessel formation. Given the heparin μislands amplify endogenous bioactive signals, this diminished effect could indicate a decrease in endogenous activity around the scaffold. Nevertheless, it was unexpected to see such a significant decrease in cell presence (DAPI signal) and implant volume at 12 months, given the lack of an engineered degradation mechanism.

### 3.4. Heparin μislands encourage early ECM deposition but all implants see decreasing ECM over time

As the ECM is a potent regulator of cell activity, we hypothesized that successful integration of endogenous ECM into MAP scaffold pores is essential for long-term stability. Although there are many proteins that comprise native dermal ECM, we chose to focus on proteins associated with new ECM deposition seen in wound healing [32–35]: fibronectin (Fn), collagen I, collagen III, and collagen IV. Fn serves as an important cell-adhesion and signaling protein in early ECM formation, while collagens I and III provide structural support as the ECM matures. Collagen IV is associated with basement membrane formation and is particularly relevant for mature vessel formation [36]. We analyzed both the overall deposition of each marker as well as deposition as a function of distance from the edge of the implant. We hypothesized that implants would have higher deposition closer to the edge and the overall deposition would increase over time.

**Fig. 4.**
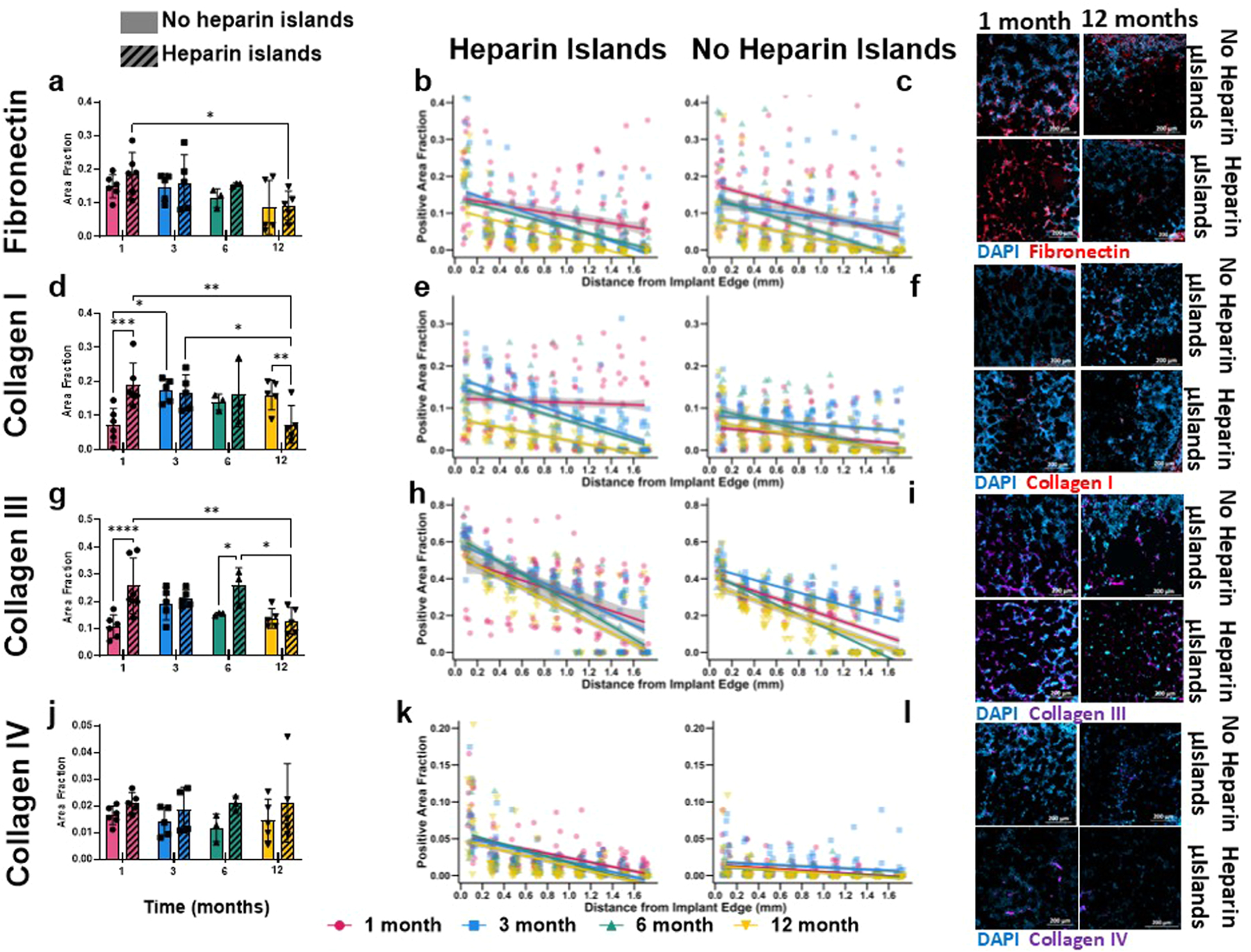
Characterizing ECM deposition. The deposition of various ECM proteins was assessed via staining and analysis of both total implant deposition (**a, d, g, j**) and deposition over radial distance (**b-c, e-f, h-i, k-l**). The four proteins analyzed and shown here are fibronectin (**a-c**), collagen I (**d-f**), collagen III (**g-i**), and collagen IV (**j-l**). Spatial analysis was performed as outlined in Section 2.7 in the Methods. Full pairwise comparison results are detailed in Table S2. Representative images were chosen from samples closest to the mean for each graph to ensure an accurate representation of the data. All scale bars are 200 μm and any white lines on images represent the edge of the implant. All statistical tests for total implant deposition are two-way repeated measures ANOVAs with Tukey post-hoc testing. *p < 0.05, **p < 0.01, ***p < 0.001, ****p < 0.0001. Error bars are mean ± SEM per group.

Fibronectin deposition is highest early on but decreases uniformly for both groups over time (Fig. 4a-c). Results from the linear model for distance yield a significant effect on the distance trend due to the interaction of heparin and time, indicating that heparin inclusion changes the distance trends for each timepoint although the overall Fn deposition is similar between groups over time. The presence of heparin μislands does facilitate increased early collagen III deposition and remodeling to collagen I but sees a decrease in collagen III at 12 months for both groups and a decrease in collagen I for Hep samples. While there is also an increase in collagen IV production in Hep samples as seen in the 2-way ANOVA results (*p* = 0.0065, heparin μislands inclusion, no statistically significant individual comparisons), the overall amount of collagen IV is markedly low compared to collagens I and III. This is expected, as it associates with maturing vessels and therefore occupies very little volume in the implant.

In addition, the distance trends show an effect of both heparin and time, with sustained collagen I deposition closer to the edge of the implant. Collagen I follows collagen III as the major structural protein in regenerating tissue following wound healing [33,37], so it is encouraging heparin μislands increases early deposition of collagen III as well as deposition and remodeling into collagen I. However, following similar trends seen in Figs. 2 and 3 with cell populations, the effect of heparin μislands diminishes at 3 and 6 months.

### 3.5. Heparin μislands encourage infiltration of CD4^+^ T-cells and additional CD68^−^ populations

Given the increase in cell activity and collagen degradation at late timepoints, we were then interested in profiling the infiltrating immune cells. Macrophages are among the first responders after implantation and their polarization towards M1-like or M2-like phenotypes is a common metric for the response to implanted biomaterials [9,38,39]. In addition, T-cells play an important role in immunity and immune responses to biomaterials [40], so characterizing T-cell infiltration was also of interest.

**Fig. 5.**
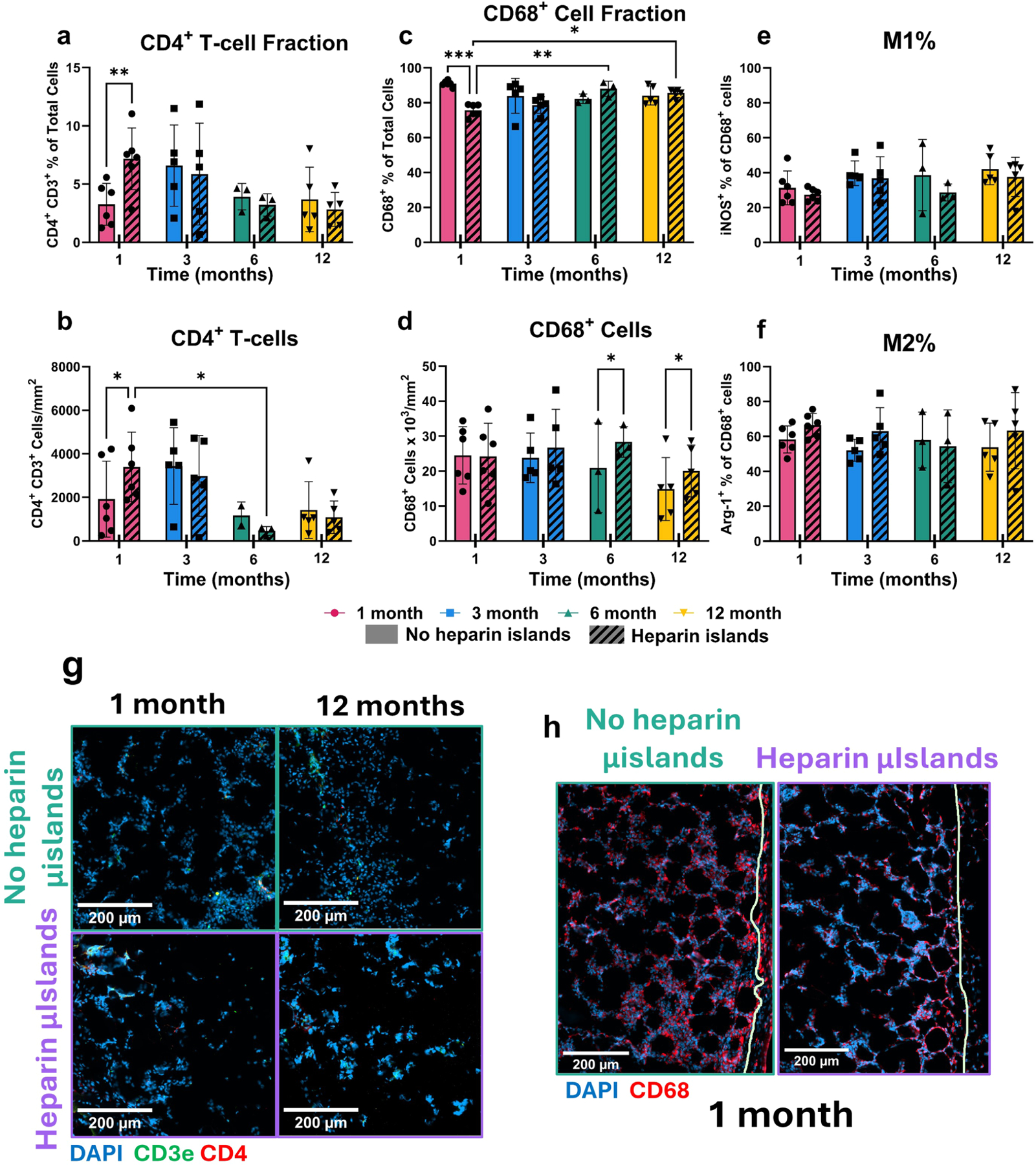
Immune cell infiltration. **a-b)** T-cell infiltration was assessed via staining for CD3e and CD4, focusing on T-helper cells. The number of CD3e^+^ CD4^+^ cells was calculated for each section then normalized to either the number of total cells **a)** or the implant area **b). c-d)** Similarly, macrophages were profiled via CD68 expression, with the number of CD68^+^ cells normalized to either the total cell count **c)** or implant area **d). e-f)** Macrophage polarization results via Arg-1 (M2-like) and iNOS (M1-like) staining. Representative images for T-cell staining (CD3e/CD4) **g)** and macrophage (CD68) staining **h)** were chosen from samples closest to the mean for each graph to ensure an accurate representation of the data. All scale bars are 200 μm and any white lines on images represent the edge of the implant. All statistical tests in this figure are two-way repeated measures ANOVAs with Tukey post-hoc testing. *p < 0.05, **p < 0.01, ***p < 0.001, ****p < 0.0001. Error bars are mean ± SEM per group.

Heparin μisland inclusion encourages increased CD4^+^ *T*-cell (CD3e^+^) cell infiltration at 1 month, but this effect decreases over time as both groups have a diminished T-cell presence (Fig. 5a-b). Like results seen in other cell types, the effect of heparin μislands is negligible after 3 months. The T-cell population decreases over time as well, trending downward in terms of total cell fraction as well as cell count normalized to area.

Hep samples at 1 month have a lower fraction of macrophages present in the implant, while the number of macrophages normalized to area is constant (Fig. 5c-d); this result suggests that heparin μislands are encouraging the infiltration of more diverse cell populations beyond the initial influx of macrophages. This fraction rises to ~85 % in both samples at 6 and 12 months but likely includes some fibroblast subpopulations, as CD68 has published cross-reactivity with certain fibroblast populations, particularly in inflammatory environments [41,42]. Additionally, given the high cell density in many samples, appropriately segmenting adjacent CD68^+^ cells was challenging. Heparin μislands not only facilitate increased cell infiltration, but selectively encourage the infiltration of non-macrophage cells (i.e., endothelial cells, T-cells, etc.) compared to NoHep samples at 1 month (Fig. 5c). Macrophage polarization was also assessed via iNOS and Arginase-1 staining for M1-like and M2-like phenotypes, respectively (Fig. 5e-f) [43]. Both groups maintained predominantly M2-like populations, and there were no significant differences between Hep and NoHep samples at any timepoints, which aligns with previous results in subcutaneous implants [14].

### 3.6. Transcriptomic analysis suggests re-activation of immune system at late timepoints

**Fig. 6.**
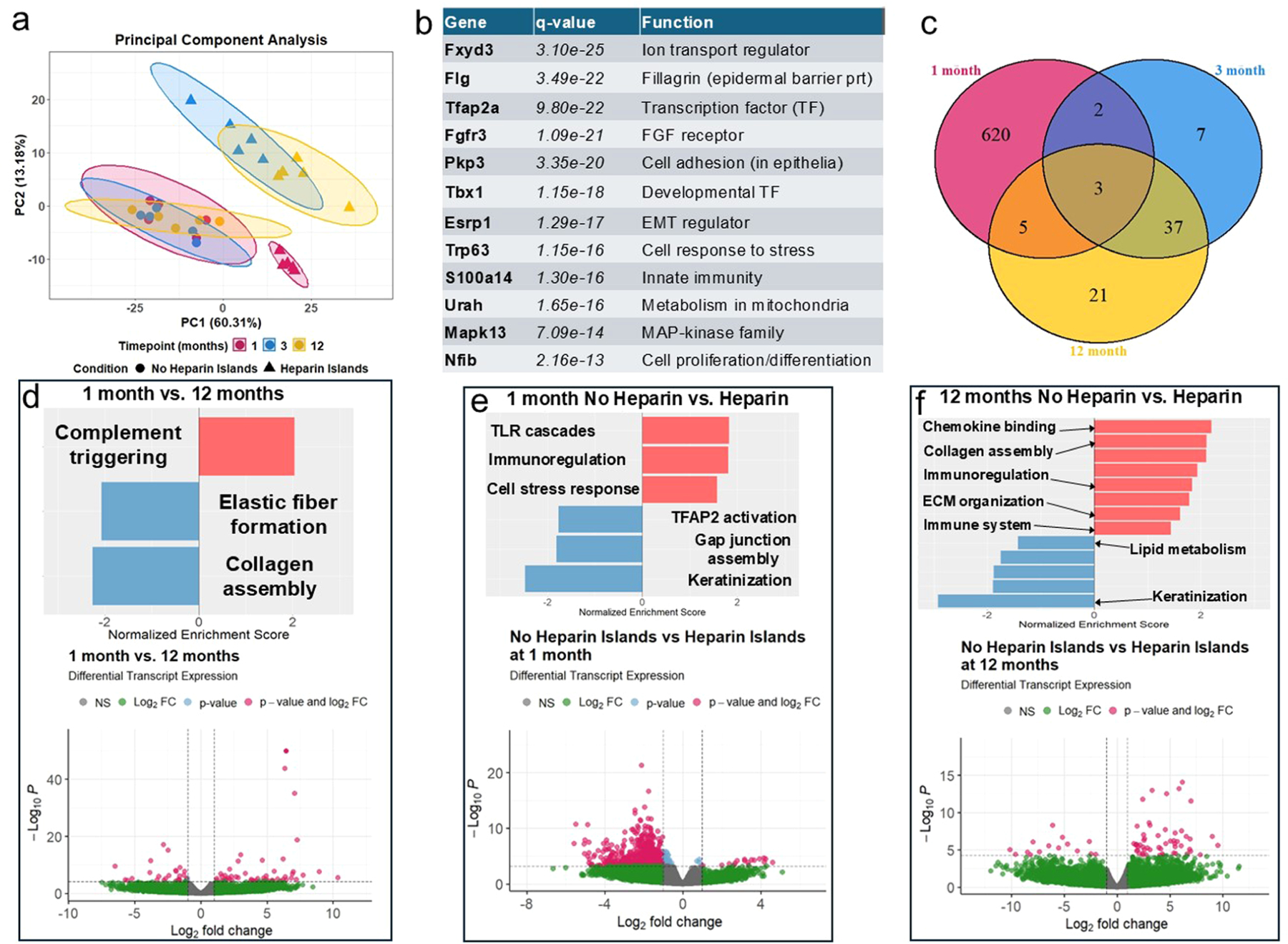
Bulk transcriptomics of implants over time. Bulk RNA-seq was performed on RNA isolated from implants at 1, 3, and 12 months. Note, all directional comparisons list the base group first (i.e., red pathways in **d)** are upregulated in 12-month samples compared to 1-month samples). **a)** Principal component analysis (PCA) of the top five hundred differentially expressed genes in the likelihood ratio test and the associated clusters, graphed using PC1 and PC2 (% variance explained included in axis title). **b)** List of the most significantly differentially expressed genes in the linear model (i.e., not specific to certain contrasts), their associated q-values, and a brief description of their function. Abbreviations: EMT (epithelial to mesenchymal transition), TF (transcription factor), prt (protein), FGF (fibroblast growth factor), MAP (mitogen-activated protein). **c)** A Venn diagram showing genes differentially expressed between Hep and NoHep samples for given timepoints. Genes in the center represent genes differentially expressed between these two groups across all three timepoints analyzed. d-**f)** Specific contrasts investigated using Wald tests in Sleuth. **d)** The effects of time, regardless of treatment (Hep vs. NoHep), were analyzed first. Comparing 1-month samples to 12-month samples yielded three pathways significantly enriched (left) as determined via GSEA and the volcano plot on the right (q-value cutoff of 0.05, log-fold change cutoff of 2). **e-f)** Comparing Hep and NoHep at 1 month **e)** and 12 months **f)** to determine what pathways are significantly up- or down-regulated (top) and the number of genes significantly differentially expressed (volcano plots, bottom). Note, pathway titles were abbreviated for clarity, full titles are included in Table S3. Full results for **f)** are included in Fig. S5 for clarity.

After isolating RNA from serial sections of the embedded implants, paired-end sequencing was performed and analyzed using the Kallisto-Sleuth pipeline [23,24] for differential gene expression analysis. Details of this analysis are in Section 2.7 of Methods. We first created a linear mixed model with random effects (i.e., batch) and predictors (i.e., timepoint). Comparing results from this full model to a reduced model accounting for only the random effects with a likelihood ratio test yielded a list of significant genes (top twelve shown in Fig. 6b) that had variability better explained by the full model. Using this full model, we identified several relevant contrasts to explore and understand specific differences over time and between Hep and NoHep samples.

Using the top five hundred differentially expressed genes from the likelihood ratio model, principal component analysis (PCA) was performed to evaluate sample clustering (Fig. 6a). The NoHep samples show little change over time while the Hep samples at 1 month appear quite different than the later timepoint clusters. Although there are many genes differentially expressed between Hep and NoHep samples at 1 month, at 3 months these differences diminish (Fig. 6c) and show very few differences. Pathway analysis for 3-months samples (compared to other timepoints and groups within 3 months) is not included in these visualizations because there were no significantly enriched pathways associated with these analyses. Volcano plots for comparisons between Hep and NoHep samples at 3 months as well as comparisons between 3 months and the other timepoints are included in the supplemental information (Fig. S4). At 1 month, where previous results show a significant effect due to the heparin μislands, Hep samples see significant enrichment of pathways involved in immune signaling and crosstalk, pointing to increased cell activity and immune response (Fig. 6e). However, this immune response does not yield a chronic response akin to the FBR, given the sustained cell infiltration and lack of fibrous capsule formation.

Regardless of heparin μisland inclusion, 12-month samples saw significantly decreased expression of genes involved in collagen and elastic fiber formation (Fig. 6d) which corroborates earlier results showing decreased ECM presence at 12 months (Fig. 4). In addition, 12-month samples have significant upregulation of genes involved in complement activation and the immune response (Fig. 6d); these genes included *Fcna* (ficolin a) and *Ighg1* (immunoglobulin heavy constant gamma 1). To explore these results further, we looked specifically at genes differentially expressed between Hep and NoHep samples at 12 months (Fig. 6f). 12-month Hep samples see an additional increase in inflammatory markers, including S100A8/A9 and IL-1β, and protease activity (MMP3, MMP9), consistent with earlier evidence of increased collagen degradation, suggesting the heparin μislands again harness the increased endogenous bioactivity.

### 3.7. Loss of porosity may re-initiate immune activation

**Fig. 7.**
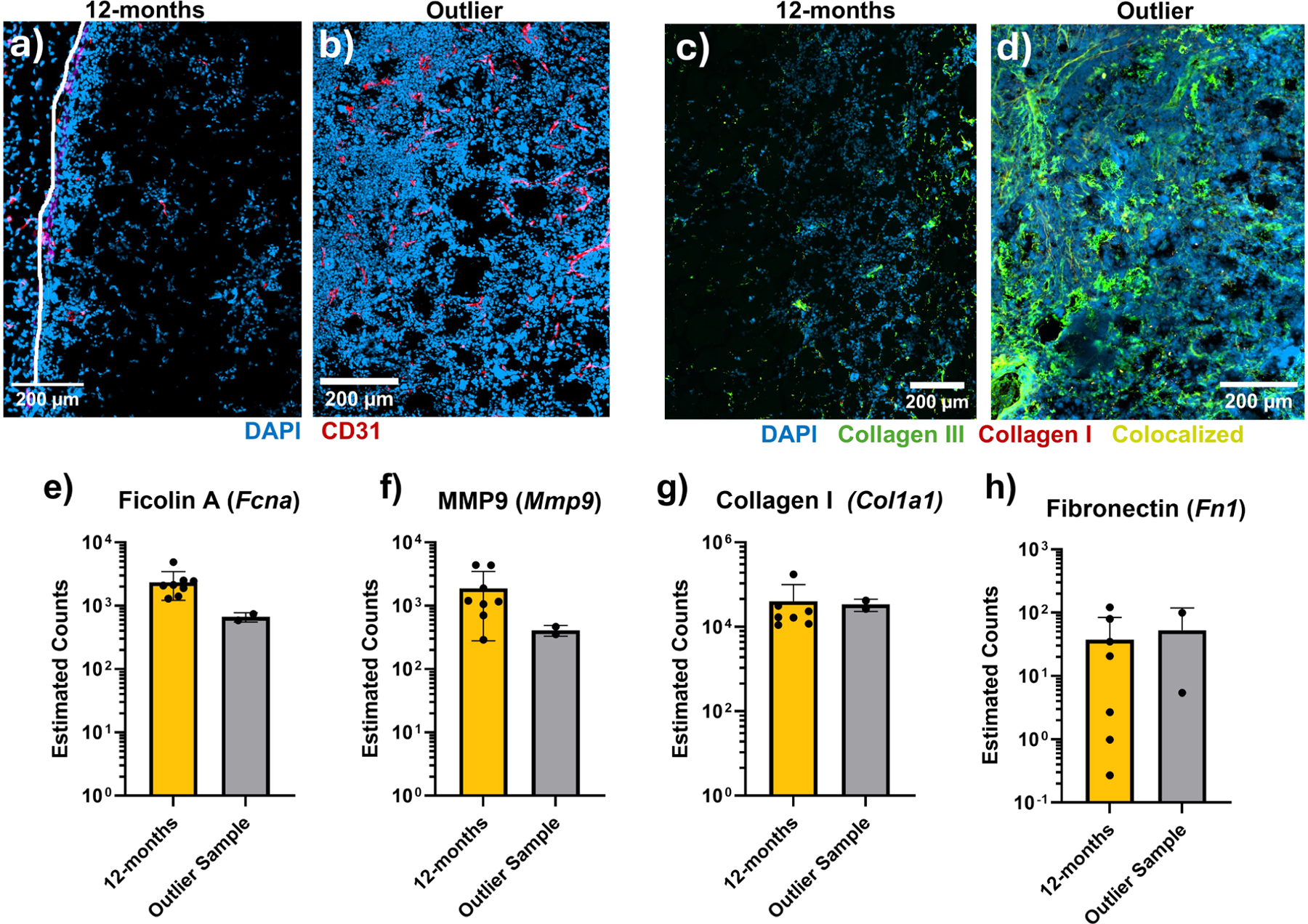
Comparison of single tissue-forming sample. **a-d)** Images of the heparin μislands-containing implant from the outlier mouse(a, c) and representative 12-month sample (also heparin μislands) (b, d) stained for DAPI and CD31^+^ endothelial cells (a, b) as well as DAPI with collagen I and III (c, d). e-h) Comparisons of normalized transcript counts from the LRT performed in Sleuth for four select genes (Fcna, Mmp2, Col1a1, and Fn1). Comparisons include both samples from the single outlier sample (both Hep and NoHep) compared with all other 12-month samples (Hep and NoHep pooled). Data is presented as estimated counts for the transcript with the highest q-value for a given gene; graphs are mean ± standard deviation.

Based on the volume loss observed in Fig. 1, we hypothesized that, to achieve tissue formation and long-term support, sufficient ECM deposition must occur to offset the loss of pore structure accompanying material degradation. Supporting this hypothesis, a single mouse in the cohort retained its height and exhibited extensive integration, vascularization, and cell proliferation compared to others; this effect was observed in both the Hep and NoHep groups. As seen in the figure below (Fig. 7), these implants had increased cell infiltration throughout the implant as well as more CD31^+^ cells and substantial ECM deposition dominated by collagen III.

Note, this sample was removed from all the analyses shown here as it was identified as an outlier in every analysis. In addition, the high cell density made the analysis in IMARIS remarkably difficult for images from this sample as the software had significant trouble segmenting cells. Rigorous differential gene analysis comparing one sample to all others is also challenging and often yields false positives, so to corroborate the results seen in Fig. 7 above, we probed specific genes within the general likelihood ratio model for this dataset and examined the normalized counts between this sample and other 12-month samples. This sample did not exhibit the same upregulation in complement-associated *Fcna* [44] and sees trends of reduced protease activity (MMP2, MMP9), although rigorous statistical analysis cannot performed. However, both implants from this mouse maintain the low-level expression of major ECM proteins, including collagen I (Col1a1) and fibronectin (Fn). There appears to be little difference between these samples and the other 12-month samples in the PCA plot (Fig. 6a), indicating that these cells are behaving significantly different at this timepoint and most of the relevant change occurred prior to this timepoint. Although we observed these trends, we do not have sufficient longitudinal data to conclude why this sample avoided the fate of the other 12-month samples. Still, this outlier sample suggests that tissue formation in the subcutaneous space is feasible using future iterations of MAP scaffolds.

## 4. Discussion

Although subcutaneous MAP scaffold implantation has previously been studied on short timescales [14,45], this study marks the longest study to-date at 12 months. Given that the microparticle formulation used in this study did not incorporate intentionally degradable cross-links, the long-term decrease in volume may be due to random hydrolysis of the PEG backbone or interactions with reactive oxygen species. Notably, hydrolysis half-lives for the relevant moieties (amides, thioethers, ethers, etc.) are measured in months to years and therefore occur very slowly; other studies have also demonstrated the stability of PEG-based hydrogels under physiological conditions on short timescales [32–34]. Additionally, the increase in volume at 1 month, while unexpected, has been observed previously [14], and is likely due to implantation-related inflammation as well as the sudden influx of interstitial fluid and cells.

Heparin μislands-containing samples experience the greatest benefit at 1 month, when this initial influx of cells and growth factors can be effectively harnessed by the heparin-containing particles as cells release growth factors and chemokines that the heparin particles can sequester. These implants appear different not only in the immunofluorescence images but also in the transcriptomic analysis, where the 1-month Hep samples appear the most different from all other groups. However, as time progress and the initial cell activity from implantation decreases, there are fewer chemotactic signals for the heparin μislands to amplify, attenuating their effect. These samples still continue to have high cell numbers near the implant edge, likely associated with the chemotactic effect of heparin μislands on cells in the surrounding tissue.

With the general influx of cells, endothelial cells (ECs) are recruited to create a vessel network to provide nutrients to migrating cells. Endogenous heparin has known interactions with vascular endothelial growth factor (VEGF) [15,16], and previous studies have shown significant vascularization within diabetic wounds with heparin μislands MAP compared to MAP without heparin [13]. Although this study was done in a relatively inert, subcutaneous environment (compared to previous studies in signal-rich inflamed wounds [13]), we still see increases in CD31^+^ cells and LECs in Hep samples. Evidence of lymph vessel formation is rare in the literature and shows how heparin μislands can facilitate the growth of functional tissue structures to support sustained cell growth and tissue formation.

Heparin also has known interactions with FGF [46], which has a positive chemotactic effect on fibroblasts and endothelial cells [47,48], providing a potential mechanism for increased fibroblast presence. Although there is not conclusive evidence of FGF-heparin interactions in this study, previous studies have shown interactions of heparin, both endogenous and conjugated to biomaterials, with VEGF and FGF [13,15,16,48]. Future studies will further interrogate the biochemical species bound to heparin in vivo, however we still propose these interactions as the primary driver of the differential response we observe. As the initial inflammation and cell migration from implantation diminishes, there are fewer signals for the heparin μislands to amplify and use to encourage additional cell activity, helping to explain the drop at longer timepoints. Additionally, heparin stability may also decrease faster than expected due to endogenous heparanases, which can be upregulated during inflammation [49,50]. Although heparin μislands affect the distribution of cells early on, a specific level of matrix protein deposition may be required to support the gel structure at late timepoints. At 12 months, the differential decrease for Hep samples in collagen I could indicate increased bioactivity and matrix degradation compared to NoHep samples, leading to accelerated degradation of collagen.

We were also interested in characterizing macrophage and T-cell infiltration into the implant via CD68, CD3e, and CD4 immunostaining. Heparin has been shown to have strong interactions with IL-2 [51], which has chemotactic effects on T-cells [52,53], among other cytokines. In addition, the increased CD68^+^ cells per area suggest the reactivation of an inflammatory response in the heparin μislands implants at late timepoints. Increased cell proliferation at late timepoints could also indicate reactivation of an inflammatory response, especially given the lack of increased stromal cell migration. However, the T-cell fraction does not significantly increase at the later timepoints, so it is unlikely this secondary immune response involves the adaptive immune response. To better understand the cellular dynamics in these implants, particularly given the results shown here, we looked to transcriptomic analysis for further insights.

Previous work has shown that a major factor in the FBR to biomaterials is complement system activation (particularly via the alternative pathway) [54], so this increase in complement-associated genes could indicate activation of the complement system at late timepoints following gradual pore collapse. This reactivation could cause further inflammation and protease activity, yielding increases in proliferation (Fig. 3f) and decreases in ECM (Fig. 4d) seen in this study. Although the alternative pathway is most often implicated in biomaterial complement activation [54], the lectin pathway (including *Fcna*) has been shown to have a limited reaction to PEG. Since the components of the alternative complement system are soluble factors produced in the liver, they will not appear in a transcriptomic analysis of local cells as performed here, but genes associated with other pathways or downstream effects will. Therefore, we suggest that the increased bioactivity from the heparin μislands may reinitiate an immune response, possibly via complement activation, and reduce scaffold stability. This contrasts with our initial hypothesis, as heparin μislands have a beneficial effect early on in this environment but potentially work against implant integration at later timepoints. Additional investigation is needed to balance pro-inflammatory and pro-regenerative pathways to achieve tissue formation without significant adverse effects.

In the single mouse that retained cells throughout the implant, we hypothesized that it facilitated sufficient ECM deposition to support the pore architecture even as the particles degraded. Given the lack of upregulation of ECM proteins in this sample, it is likely the synthesis and deposition of the proteins occurred prior to 12 months. Further investigation is still required to confirm our hypothesis, but these results suggest that the support provided by the new ECM help the implant avoid an inflammatory response. Future studies will attempt to accelerate this timeline of ECM deposition to avoid an immune response and maintain implant structure and integration. Finally, more work will be done to characterize the effect of the implant tissue environment, given the initial vocal fold study [12] was performed in atrophied muscle yet observed *de novo* tissue generation.

## 5. Conclusions

In this study, we describe the dynamics of matrix protein deposition and cell infiltration over time in MAP scaffolds with and without heparin μislands. We provide evidence for a mechanism of heparin μislands wherein they harness endogenous signals for growth and migration and facilitate the in-growth of more diverse cell populations. Additionally, we profile infiltrating cell populations at 1-, 3-, 6-, and 12-months post-implantation; both implants see a significant decrease in cell presence and structural protein deposition at 12 months. Transcriptomic analysis reveals an increase in inflammatory signals at 12 months following a long period of inactivity, with additional evidence suggesting that a collapse of pores in the gel without sufficient protein structure leads to a reactivation of the inflammatory response as the implant presents more like a nanoporous implant to the surrounding tissue and initiates the complement system alternative pathway implicated in the FBR. We also show how heparin μislands can harness and amplify endogenous bioactivity but cannot generate new signals, diminishing their effects in noninflammatory or relatively inert environments. Future studies will focus on the impact of the identity and activity of the surrounding tissue (e.g., dynamic muscle versus more static fascia) and investigating strategies to facilitate increased structural support of scaffolds via exogenous protein incorporation.

## Supplementary Material

MMC1
