# Supplementary material for: Exploration of biomaterial-tissue integration in heterogeneous microporous annealed particle scaffolds in subcutaneous implants over 12 months
Source: Acta Biomater. Author manuscript; Available in PMC 2025 Apr 4. (PMC11968225; doi:10.1016/j.actbio.2025.02.020)
Supplement: MMC1 [file NIHMS2063566-supplement-MMC1.docx]

**Supplemental Information**


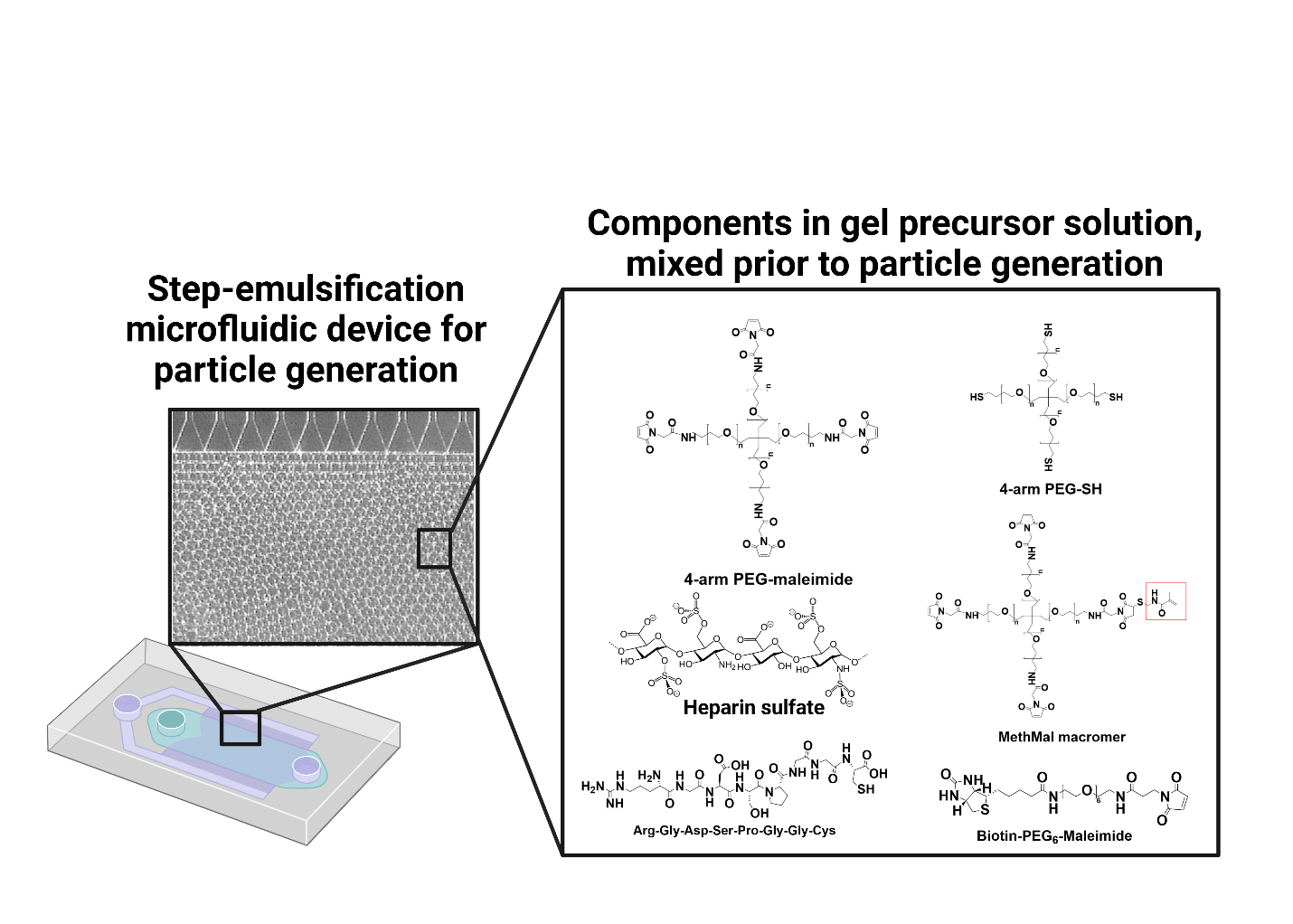


**Figure S1:** Left: Schematic of parallel-step emulsification microfluidic device used for particle generation. Right: Chemical structures of gel precursor constituents. Note, the red box on the MethMal structure highlights the methacrylamide group that participates in radical-based crosslinking to anneal adjacent particles together. The cell adhesion peptide RGD (full sequence: RGDSPGGC) is shown above with its full structure.


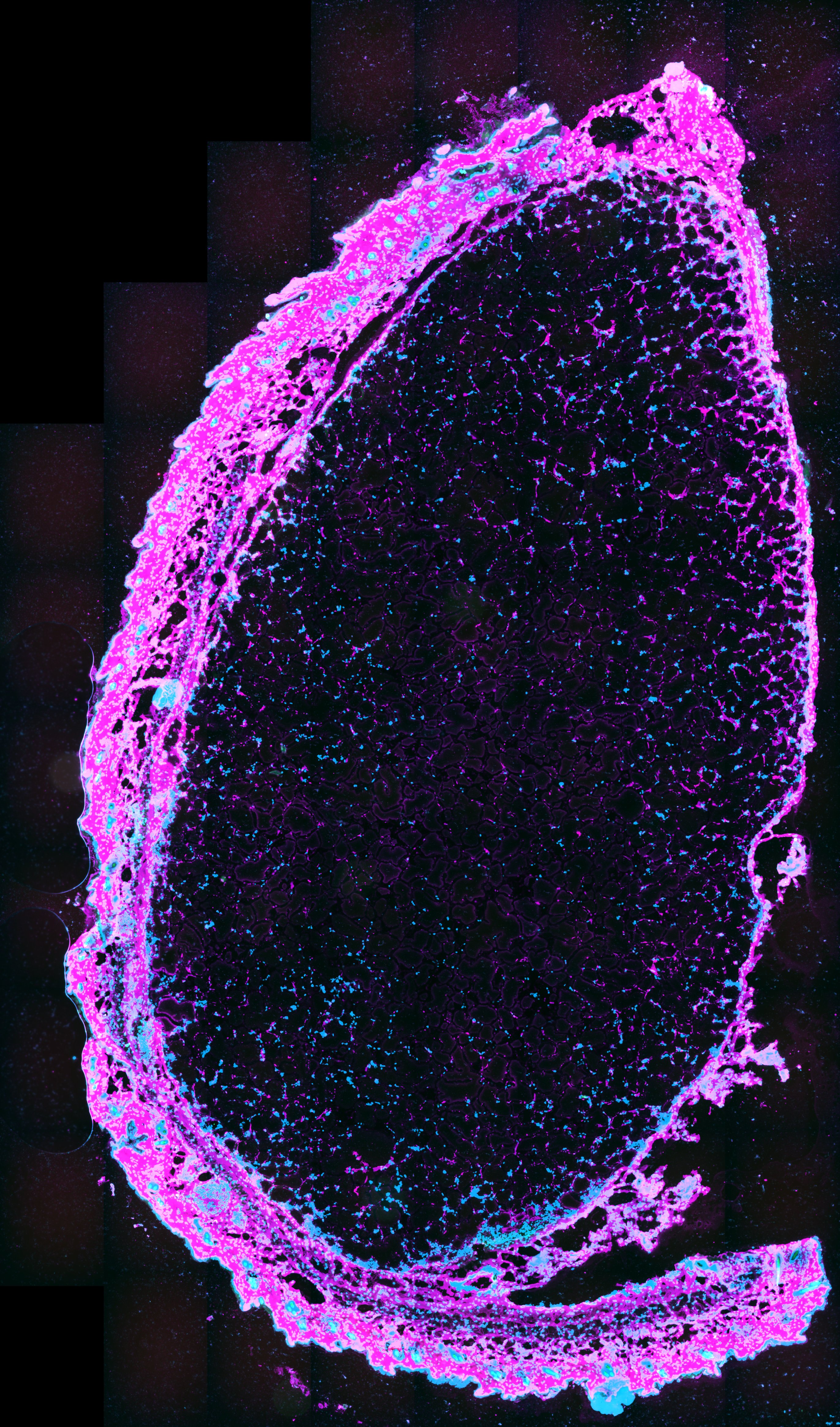

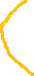

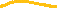

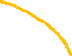

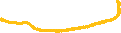

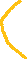

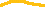

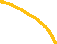

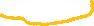

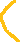

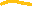

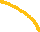

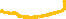

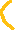

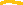

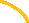

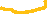

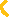

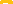

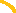

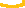

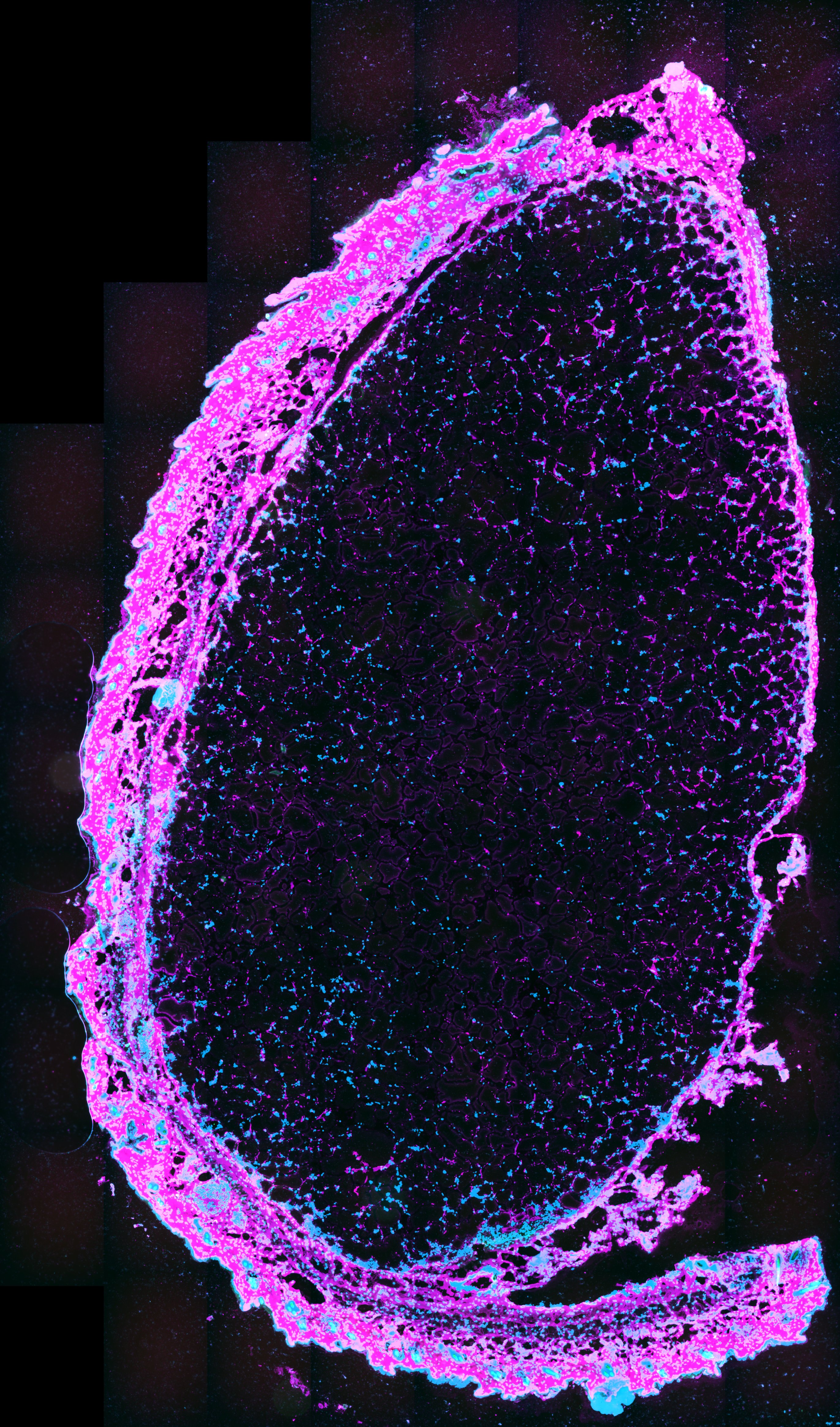

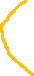

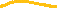

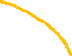

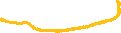


**Figure S2:** Image analysis method via concentric rings. After thresholding via the optimal algorithm (determined using the negative controls), implants were outlined in FIJI using the freehand ROI tool, then this ROI was iteratively eroded by 100 µm until the center of the implant was reached. At each point, the total pixels and number of positive pixels in each ROI was recorded. In Microsoft Excel, these values were inputted, and subsequent erosions were subtracted from each other to yield values for each ring (rather than the entire ROI) that were used to calculate the percent positive area. All code is available upon request.

**Figure S3:** Analysis of Day 0 implant measurements with calipers. All statistical tests in this figure are mixed models with Tukey post-hoc testing. *p<0.05, **p<0.01, ***p<0.001, ****p<0.0001. Error bars are mean ± SEM per group. Outliers from any of the datasets were removed using the Robust Regression and Outlier Removal (ROUT) method (Q = 1%) in GraphPad Prism.

**Figure S4:** Volcano plots representing results from comparisons between No Heparin and Heparin groups at 3 months in addition to comparing 3-months with 1- and 12-month groups.


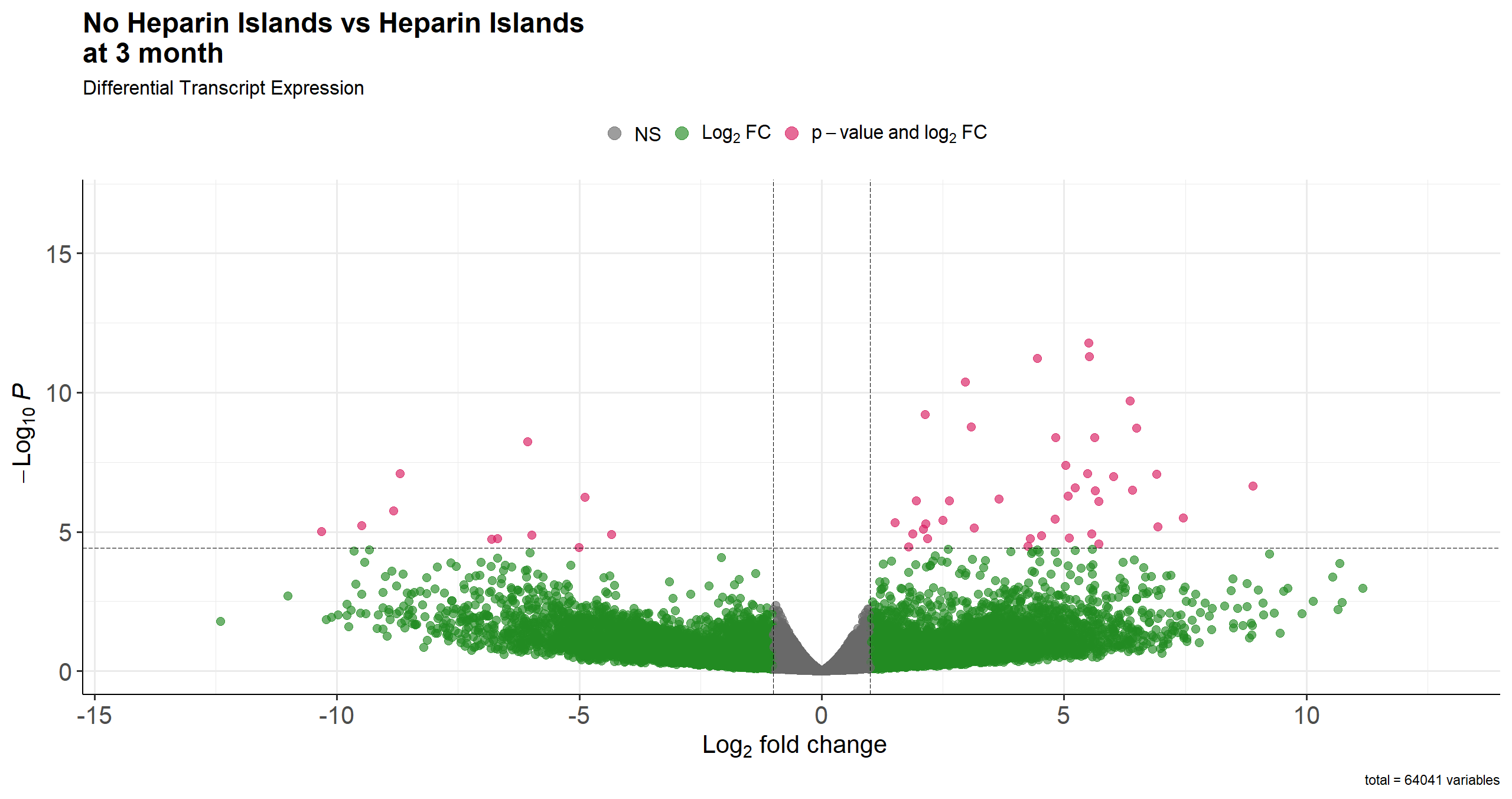

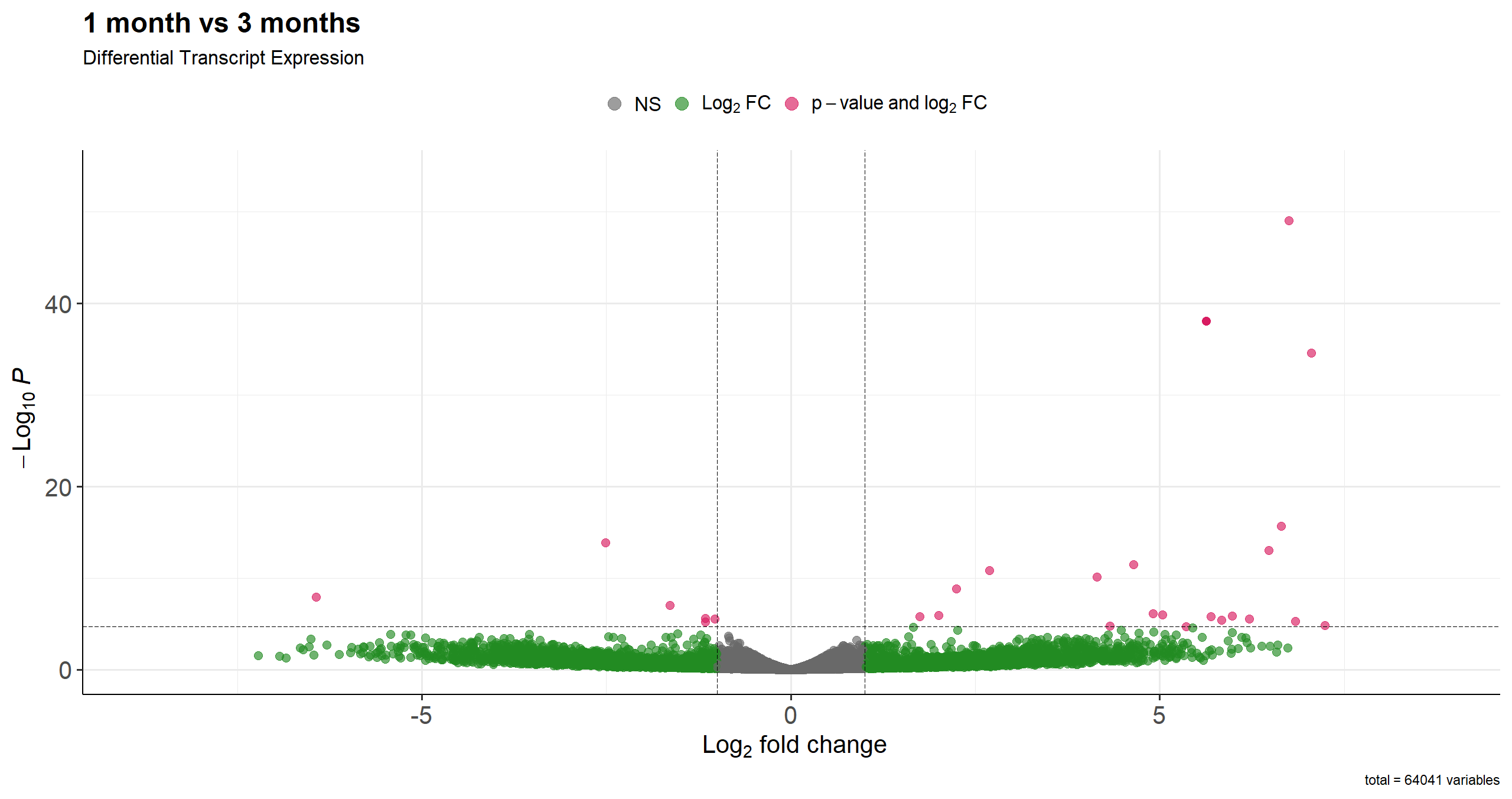


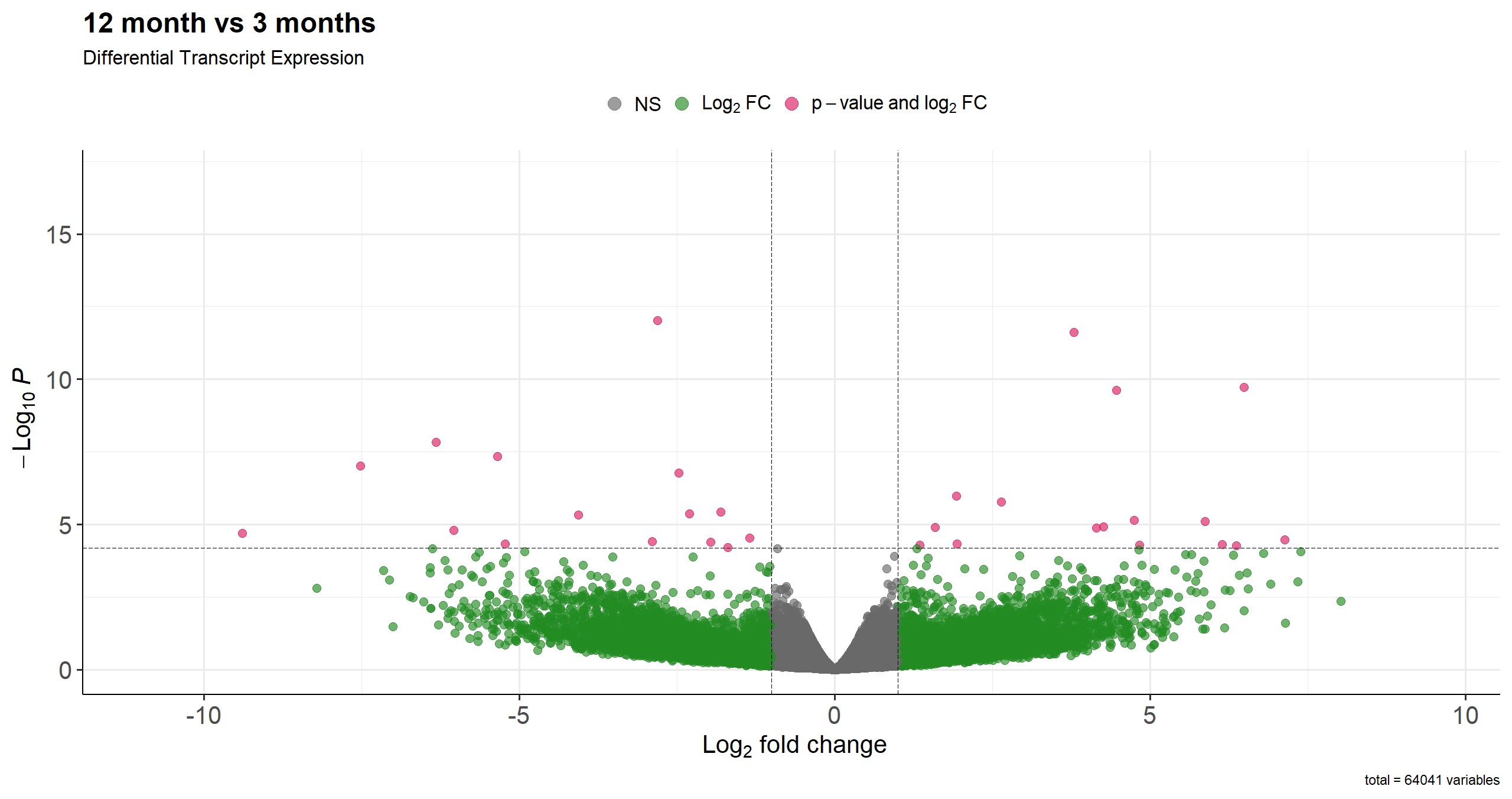


**Figure S5:** Full pathway analysis results comparing No Heparin and Heparin groups at 12 months.


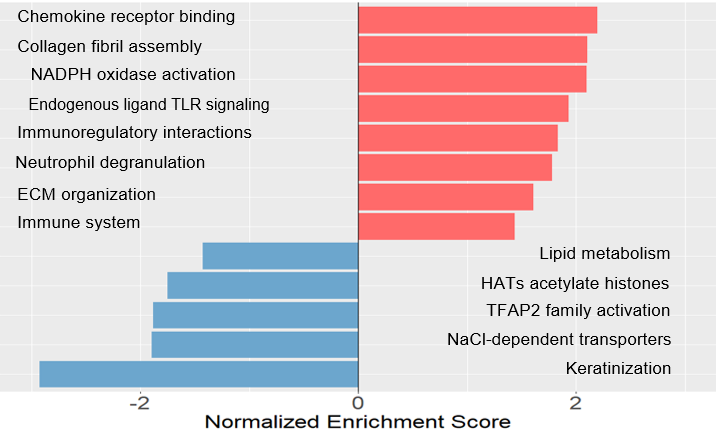


**Table S1: Antibody conditions and protocols**

| Primary Antibody | Secondary Antibody | Blocking Buffer | Fixation | TrueView |
| --- | --- | --- | --- | --- |
| Collagen I (Rockland 600-401-103.01, 1:200) | Donkey anti-rabbit AlexaFluor 647 (1:750) | 5% serum in PBS-T | Methanol (-20°C) | Yes |
| Collagen III (Novus Bio, NBP1-26547, 1:100) | Donkey anti-goat AlexaFluor 594 (1:750) | 5% serum in PBS-T | Methanol (-20°C) | Yes |
| Fibronectin (Abcam, ab2413, 1:75) | Donkey anti-rabbit AlexaFluor 594 (1:400) | 5% serum in PBS-T | Acetone (-20°C) | Yes |
| Collagen IV (Sigma-Aldrich, AB769, 1:100) | Donkey anti-goat AlexaFluor 647 (1:600) | 5% serum in PBS-T | Acetone (-20°C) | Yes |
| CD31 (BD Biosciences, #553370, 1:150) | Donkey anti-rat AlexaFluor 647 (1:750) | 10% serum in PBS-T | 4% PFA | Yes |
| Podoplanin (R&D systems, AF3244, 1:200) | Donkey anti-goat AlexaFluor 594 (1:750) | 10% serum in PBS-T | 4% PFA | Yes |
| CD68 (BioLegend, #137001, 1:75) | Goat anti-rat AlexaFluor 647 (1:500) | 5% serum in PBS-T with 1% FC-block | Acetone (-20°C) | No |
| iNOS (Cell Signaling Technology, #13120, 1:75) | Goat anti-rabbit AlexaFluor 555 (1:500) | 5% serum in PBS-T with 1% FC-block | Acetone (-20°C) | No |
| Arginase-1 (Novus Bio, NBP2-03618, 1:75) | Goat anti-mouse AlexaFluor 594 (1:500) | 5% serum in PBS-T with 1% FC-block | Acetone (-20°C) | No |
| CD3e (Abcam, ab16669, 1:75) | Goat anti-rabbit AlexaFluor 594 (1:500) | 5% serum in PBS-T with 1% FC-block | Acetone (-20°C) | No |
| CD4 (ThermoFisher, #14-0041-82, 1:50) | Goat anti-rat AlexaFluor 647 (1:500) | 5% serum in PBS-T with 1% FC-block | Acetone (-20°C) | No |
| Ki67 (Invitrogen MA5-14520, 1:400) | Donkey anti-rabbit AlexaFluor 555 (1:500) | 5% serum in PBS-T with 1% FC-block and 2% BSA | 4% PFA | No |
| TE-7 (Novus Bio, NVP2-50082, 1:150) | Donkey anti-mouse AlexaFluor 647 (1:750) | 5% serum in PBS-T with 1% FC-block and 2% BSA | 4% PFA | No |

**Table S2: p-values for multiple comparisons of DAPI and ECM marker slopes**

Note: green cells indicate p ≤ 0.05.

*DAPI*

| Group | Timepoint | Comparison | p-values |
| --- | --- | --- | --- |
| Heparin | . | 1 month - 3 month | 2.88E-04 |
| Heparin | . | 1 month - 6 month | 5.37E-06 |
| Heparin | . | 1 month - 12 month | 2.46E-03 |
| Heparin | . | 3 month - 6 month | 4.89E-01 |
| Heparin | . | 3 month - 12 month | 8.82E-01 |
| Heparin | . | 6 month - 12 month | 1.56E-01 |
| No-Heparin | . | 1 month - 3 month | 9.56E-01 |
| No-Heparin | . | 1 month - 6 month | 4.18E-03 |
| No-Heparin | . | 1 month - 12 month | 3.46E-01 |
| No-Heparin | . | 3 month - 6 month | 8.05E-04 |
| No-Heparin | . | 3 month - 12 month | 1.26E-01 |
| No-Heparin | . | 6 month - 12 month | 1.63E-01 |
| . | 1 month | Heparin - (No-Heparin) | 5.98E-01 |
| . | 3 month | Heparin - (No-Heparin) | 2.47E-07 |
| . | 6 month | Heparin - (No-Heparin) | 6.14E-02 |
| . | 12 month | Heparin - (No-Heparin) | 8.93E-03 |

*Fibronectin*

| Group | Timepoint | Comparison | p-values |
| --- | --- | --- | --- |
| Heparin | . | 1 month - 3 month | 2.00E-02 |
| Heparin | . | 1 month - 6 month | 4.97E-01 |
| Heparin | . | 1 month - 12 month | 3.13E-01 |
| Heparin | . | 3 month - 6 month | 6.58E-01 |
| Heparin | . | 3 month - 12 month | 6.68E-01 |
| Heparin | . | 6 month - 12 month | 9.99E-01 |
| No-Heparin | . | 1 month - 3 month | 1.09E-01 |
| No-Heparin | . | 1 month - 6 month | 9.12E-01 |
| No-Heparin | . | 1 month - 12 month | 6.18E-01 |
| No-Heparin | . | 3 month - 6 month | 5.03E-02 |
| No-Heparin | . | 3 month - 12 month | 7.67E-01 |
| No-Heparin | . | 6 month - 12 month | 3.41E-01 |
| . | 1 month | Heparin - (No-Heparin) | 4.25E-02 |
| . | 3 month | Heparin - (No-Heparin) | 2.27E-03 |
| . | 6 month | Heparin - (No-Heparin) | 4.29E-01 |
| . | 12 month | Heparin - (No-Heparin) | 3.19E-01 |

*Collagen I*

| Group | Timepoint | Comparison | p-values |
| --- | --- | --- | --- |
| Heparin | . | 1 month - 3 month | 1.32E-08 |
| Heparin | . | 1 month - 6 month | 5.40E-05 |
| Heparin | . | 1 month - 12 month | 1.85E-03 |
| Heparin | . | 3 month - 6 month | 7.54E-01 |
| Heparin | . | 3 month - 12 month | 1.50E-02 |
| Heparin | . | 6 month - 12 month | 4.15E-01 |
| No-Heparin | . | 1 month - 3 month | 1.00E+00 |
| No-Heparin | . | 1 month - 6 month | 9.82E-03 |
| No-Heparin | . | 1 month - 12 month | 3.93E-01 |
| No-Heparin | . | 3 month - 6 month | 1.07E-02 |
| No-Heparin | . | 3 month - 12 month | 4.12E-01 |
| No-Heparin | . | 6 month - 12 month | 2.37E-01 |
| . | 1 month | Heparin - (No-Heparin) | 2.73E-01 |
| . | 3 month | Heparin - (No-Heparin) | 4.89E-08 |
| . | 6 month | Heparin - (No-Heparin) | 5.46E-01 |
| . | 12 month | Heparin - (No-Heparin) | 2.03E-01 |

*Collagen III*

| Group | Timepoint | Comparison | p-values |
| --- | --- | --- | --- |
| Heparin | . | 1 month - 3 month | 3.91E-03 |
| Heparin | . | 1 month - 6 month | 2.30E-07 |
| Heparin | . | 1 month - 12 month | 3.50E-07 |
| Heparin | . | 3 month - 6 month | 4.42E-02 |
| Heparin | . | 3 month - 12 month | 2.05E-01 |
| Heparin | . | 6 month - 12 month | 7.32E-01 |
| No-Heparin | . | 1 month - 3 month | 3.63E-01 |
| No-Heparin | . | 1 month - 6 month | 1.19E-02 |
| No-Heparin | . | 1 month - 12 month | 6.81E-01 |
| No-Heparin | . | 3 month - 6 month | 5.00E-05 |
| No-Heparin | . | 3 month - 12 month | 2.56E-02 |
| No-Heparin | . | 6 month - 12 month | 1.20E-01 |
| . | 1 month | Heparin - (No-Heparin) | 9.63E-01 |
| . | 3 month | Heparin - (No-Heparin) | 5.53E-07 |
| . | 6 month | Heparin - (No-Heparin) | 2.51E-02 |
| . | 12 month | Heparin - (No-Heparin) | 4.28E-06 |

*Collagen IV*

| Group | Timepoint | Comparison | p-values |
| --- | --- | --- | --- |
| Heparin | . | 1 month - 3 month | 7.96E-01 |
| Heparin | . | 1 month - 6 month | 1.48E-01 |
| Heparin | . | 1 month - 12 month | 7.75E-01 |
| Heparin | . | 3 month - 6 month | 6.36E-01 |
| Heparin | . | 3 month - 12 month | 1.00E+00 |
| Heparin | . | 6 month - 12 month | 6.32E-01 |
| No-Heparin | . | 1 month - 3 month | 9.72E-01 |
| No-Heparin | . | 1 month - 6 month | 9.92E-01 |
| No-Heparin | . | 1 month - 12 month | 9.99E-01 |
| No-Heparin | . | 3 month - 6 month | 9.99E-01 |
| No-Heparin | . | 3 month - 12 month | 9.52E-01 |
| No-Heparin | . | 6 month - 12 month | 9.82E-01 |
| . | 1 month | Heparin - (No-Heparin) | 3.29E-06 |
| . | 3 month | Heparin - (No-Heparin) | 3.23E-07 |
| . | 6 month | Heparin - (No-Heparin) | 4.49E-08 |
| . | 12 month | Heparin - (No-Heparin) | 2.47E-06 |

**Table S3: Reactome pathway analysis abbreviations**

| **Abbreviated Title** | **Full Reactome Title** |
| --- | --- |
| Complement triggering | Initial triggering of complement |
| Elastic fiber formation | Elastic fibre formation |
| Collagen assembly | Collagen formation |
| TLR cascades | Toll-like receptor cascades |
| Immunoregulation | Immunoregulatory interactions between a lymphoid and non-lymphoid cell |
| Cell response to chemical stress | Cellular response to chemical stress |
| TFAP2 family activation | Activation of the TFAP (AP2) family of transcription factors |
| Gap junction assembly | Gap junction assembly |
| Keratinization | Keratinization |
| Chemokine receptor binding | Chemokine receptors bind chemokines |
| Collagen fibril assembly | Assembly of collagen fibrils and other multimeric structures |
| NADPH oxidase activation | Rho GTPases activate NADPH oxidases |
| Endogenous ligand TLR signaling | Regulation of TLRs by endogenous ligands |
| Neutrophil degranulation | Neutrophil degranulation |
| ECM organization | Extracellular matrix organization |
| Immune system | Immune system |
| Lipid metabolism | Metabolism of lipids |
| HATs acetylate histones | HATs acetylate histones |
| Nacl-dependent transporters | NaCl dependent neurotransmitter transporters |
